# Reactivity of the Asymmetric Wells-Dawson Ion: Lanthanide-Containing 34-Tungsto-2-Phosphates [Ln(P(H_4_)W_17_O_61_)_2_]^19–^ (Ln = La^3+^, Ce^3+^, Eu^3+^, Gd^3+^, Yb^3+^, Lu^3+^, Y^3+^)

**DOI:** 10.1021/acs.inorgchem.5c04807

**Published:** 2026-01-02

**Authors:** Mahmoud Elcheikh Mahmoud, Bassem S. Bassil, Anupam Sarkar, Ji-o Kim, Senthil Kumar Kuppusamy, Concepción Molina-Jirón, Eufemio Moreno-Pineda, Nikoleta Malinova, Appu Sunil, Wolfgang Wernsdorfer, Mario Ruben, Ulrich Kortz

**Affiliations:** † School of Science, 84498 Constructor University, Campus Ring 1, 28759 Bremen, Germany; ‡ Institute of Quantum Materials and Technologies (IQMT), 150232 Karlsruhe Institute of Technology (KIT), Kaiserstraße 12, D-76131 Karlsruhe, Germany; § Facultad de Ciencias Naturales, Exactas y Tecnología, Depto. de Bioquímica, Universidad de Panamá, Panamá 0824, Panama; ∥ Facultad de Ciencias Naturales, Exactas y Tecnología, Grupo de Investigación de Materiales, Universidad de Panamá, Panamá 0824, Panama; ⊥ Facultad de Ciencias Naturales, Exactas y Tecnología, Depto. de Química-Física, Universidad de Panamá, 0824 Panamá, Panama; # Physikalisches Institut, Karlsruhe Institute of Technology (KIT), Kaiserstraße 12, D-76131 Karlsruhe, Germany; ¶ Institute of Nanotechnology (INT), Karlsruhe Institute of Technology (KIT), Kaiserstraße 12, D-76131 Karlsruhe, Germany; ∇ Faculty of Chemical and Food Technology, Department of Inorganic Chemistry, Slovak University of Technology, Radlinského 2101/9, 812 37 Bratislava, Slovakia; ○ Centre Européen de Sciences Quantiques (CESQ), Institut de Science et d’Ingénierie Supramoléculaires (ISIS), 8 allée Gaspard Monge, BP 70028, 67083 Strasbourg Cedex, France

## Abstract

We report on the synthesis of the lanthanide-containing 17-tungsto-1-phosphates [Ln­(P­(H_4_)­W_17_O_61_)_2_]^19–^ (Ln = La^3+^, Ce^3+^, Eu^3+^, Gd^3+^, Yb^3+^, Lu^3+^, Y^3+^), comprising a lanthanide ion connecting two [P­(H_4_)­W_17_O_61_]^11–^ units in a *syn*-configuration. The compounds were characterized in the solid state by IR, powder XRD, and TGA, and in solution by ^31^P and ^183^W NMR. Alternating current magnetic susceptibility investigations revealed the Ce^3+^ and Yb^3+^ analogues to be single-molecule magnets (SMM), which was further corroborated by sub-kelvin temperature μSQUID studies. For the Eu^3+^ analogue, we have observed a strong ^5^D_0_ → ^7^F_*J*_ (*J* = 0–4) emission along with a weaker ^5^D_*J*_ (*J* = 1, 2, or 3) → ^7^F_*J*_ emission arising from higher excited state manifolds, upon excitation via the ^7^F_0_ → ^5^L_6_ transition at 395 nm. Analysis of the steady-state and time-resolved data suggests a distorted square-antiprismatic coordination geometry around the Eu^3+^ center. The presence of water molecules residing in the outer coordination sphere appears to decrease the intrinsic quantum yield (φ_Eu_) by providing O–H oscillators as a nonradiative relaxation channel. The observed branching ratio of about 41% for the ^5^D_0_ → ^7^F_4_ transition highlights that [Eu­(P­(H_4_)­W_17_O_61_)_2_]^19–^ exhibits a pronounced ^5^D_0_ → ^7^F_4_ emission.

## Graphical abstract

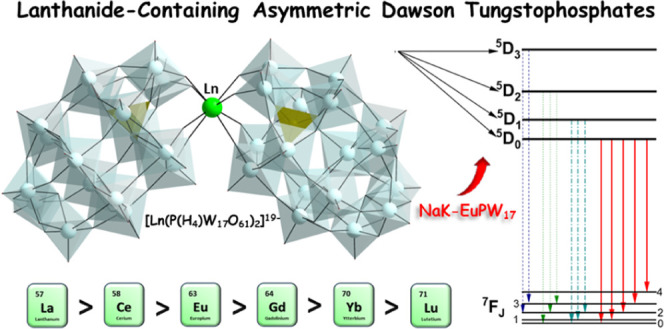

## Introduction

Polyoxometalates (POMs) are anionic metal-oxo anions comprising early transition metal addenda in high oxidation states, such as W^VI^, Mo^VI^, and V^V^. The class of POMs exhibits a manifold of physicochemical properties due to their discrete, anionic nature, resembling soluble fragments of extended metal oxides, oxygen-rich surfaces, tunable charge density, acid strength, redox potentials, and chemical composition.^1^ POMs include a wide structural range, with various sizes and shapes, providing a solid basis for molecular design and assembly to synthesize target molecular aggregates and functional materials.^2^ Lacunary (vacant) POMs can be obtained by the removal of one, two, or three MO_6_ units from the plenary structures leading to the formation of mono, di, and trilacunary polyanion derivatives.^1^ Such lacunary POMs can be considered as inorganic polydendate ligands which can coordinate to oxophilic guests such as d-block metal ions, lanthanide and actinide ions, resulting in polyanions with attractive properties and potential applications.^3^ Lanthanide ions are larger than d-block metal ions and hence cannot be fully accommodated in the POM vacancy, and as a result, they act frequently as linkers of lacunary POM units, resulting in dimers, trimers, etc., or even extended lattices. Such compounds and materials broaden the scope of physicochemical properties.^4^

In 1953, Dawson reported the crystal structure of the polyanion known nowadays as Wells-Dawson ion with the general formula [X_2_M_18_O_62_]^6–^ (X = P, As; M = Mo, W).^5^ This structure can be viewed as being formed via fusion of two trilacunary [*A*-XM_9_O_34_]^9–^ ions at the lacunary sites. This linkage mode results in two “belts” of six addenda each, and two trinuclear “caps” at each end. It is worth noting that upon reduction, the extra electrons reside mainly in the belt positions of the Wells-Dawson ion, which impacts the chemical properties of the structure.^1,6^ In addition, each XO_4_ heterogroup is linked to the M_3_O_13_ cap via a μ_4_-oxo bridge and to the six belt octahedra via three μ_3_-oxo bridges. In 2000 an interesting advance in the chemistry of Wells-Dawson-type POMs emerged when Contant and co-workers reported the synthesis and solution ^31^P and ^183^W NMR spectra of the “asymmetric” Wells-Dawson ion [P­(H_4_)­W_18_O_62_]^7–^, along with its monovacant derivative [P­(H_4_)­W_17_O_61_]^11–^, and the group suggested the vacant site to be adjacent to the phosphorus heteroatom.^7a^ In 2001 the same group introduced the arsenic­(V)-analogue [As­(H_4_)­W_18_O_62_]^7–^, alongside the tetra-zinc­(II)-containing sandwich-typepolyanions [Zn_4_(H_2_O)_4_(As­(H_4_)­W_15_O_56_)_2_]^18–^ and [Cu_4_(H_2_O)_2_(As­(H_4_)­W_15_O_56_)_2_]^18–^, which can be seen as asymmetric analogues of the Weakley-dimer, and these polyanions were studied for their electrocatalytic activity toward nitrite and nitrate reduction.^7b^ In 2003 Pope and co-workers refined the synthetic protocols and provided structural comparisons between the asymmetric [As­(H_4_)­W_18_O_62_]^7–^ and the symmetrical [As_2_W_18_O_62_]^6–^ species.^7c^ This work was further extended by Mbomekalle et al., who explored the redox behavior and transition metal functionalization of the plenary [P­(H_4_)­W_18_O_62_]^7–^ species and confirmed the viability of the monovacant [P­(H_4_)­W_17_O_61_]^11–^ for further derivatization.^7d^ To date, the only nondisordered crystallographically characterized asymmetric monolacunary Wells-Dawson ion is the Ce^III^-containing polyanion [Ce^III^{X­(H_4_)­W_17_O_61_}_2_]^19–^ (X = P^V^, As^V^), reported by Pope and co-workers in 2005, which offered valuable insights into the internal protonation features and confirmed the position of the P or As heterogroup to be adjacent to the lacunary site.^7e^ In contrast to the numerous examples of lanthanide-substituted polyanions containing the symmetrical [P_2_W_17_O_61_]^10–^ unit, the asymmetric derivative [P­(H_4_)­W_17_O_61_]^11–^ remains largely unexplored in terms of its versatility as an inorganic ligand toward *f*-block metal ions.^7^ Lanthanide ions are known to form mainly 1:2 assemblies with monovacant Keggin and (symmetrical) Wells-Dawson polyanions, with the lanthanide ion usually being 8-coordinated in a square-antiprismatic fashion.^8^ The incorporation of lanthanide ions into POM frameworks not only enriches the structural diversity but also opens possibilities for interesting physicochemical properties. In particular, lanthanide-based POMs have emerged as promising candidates for single-molecule magnets (SMMs) due to their large magnetic anisotropy, as well as for photoluminescent applications arising from their characteristic 4f–4f electronic transitions.^9,10^ A systematic investigation of *f*-block metal ion-substituted derivatives of the monovacant [P­(H_4_)­W_17_O_61_]^11–^ polyanion is warranted, as to date only one crystallographically characterized derivative has been reported in the literature.^7e^ Herein, we report on the synthesis, structural characterization of a complete series of asymmetric Wells-Dawson-type POMs incorporating various lanthanide metal ions, and a study of their magnetic and luminescence properties.

## Experimental Section

### Instrumentation

All chemicals were used as received without any additional purification. Infrared (FT-IR) spectra of solid-state samples were recorded using KBr pellets on a Nicolet Avatar 370 spectrophotometer, operating over the range of 400–4000 cm^–1^ with a resolution of 4 cm^–1^ and 32 scans per spectrum. Peak intensities are shown as follows: w (weak), m (medium), s (strong), and sh (shoulder). Thermogravimetric analysis (TGA) was performed using a TA Instruments SDT Q600 under a nitrogen atmosphere, with samples heated from room temperature to 600 °C at a rate of 5 °C/min. Multinuclear NMR spectroscopy was conducted on a JEOL ECS 400 MHz instrument, employing a 5 mm probe for ^31^P and a 10 mm probe for ^183^W nuclei. Elemental analysis (Na, K, P, W, and Ln) was determined at the Zentrallabor of Technische Universität Hamburg (TUHH), Am Schwarzenberg-Campus 1, 21073 Hamburg, Germany. Single-crystal X-ray diffraction data were collected on a Rigaku XtaLAB Synergy Dualflex HyPix diffractometer equipped with a kappa geometry goniometer and a graphite monochromator (λ = 0.71073 Å, MoKα radiation). Crystals were mounted on Hampton cryoloops using Paratone-N oil and measured at 100 K. Data collection and indexing were performed using the CrysAlisPro software package.^10a^ Empirical absorption corrections were applied using the ABSPACK program.^10b^ Structures were solved via direct methods, followed by successive difference Fourier map analyses. Refinements were carried out using SHELXL-2014 against all data by full-matrix least-squares methods on |*F*|,^2^ with anisotropic displacement parameters applied to all non-hydrogen atoms.^10c^ Crystal structure illustrations were generated using Diamond, version 3.2 (Crystal Impact GbR). The crystallographic data are summarized in Table 1. Powder X-ray diffraction (PXRD) data were acquired on a Rigaku Miniflex 600 (Rigaku Corporation, Tokyo, Japan) using a primary beam Cu Kα radiation (λ = 1.541838 Å) at 40 kV and 15 mA. The instrument scanning 2θ range was from 3° to 40° in steps of 0.02°, with a scan speed of 5°/min.

**Table 1:** Single-Crystal XRD Data and Structure Refinement for NaK-LnPW_17_

compound	**NaK-LaPW_17_**	**NaK-CePW_17_**	**NaK-EuPW_17_**	**NaK-GdPW_17_**	**NaK-YbPW_17_**	**NaK-LuPW_17_**	**NaK-YPW_17_**
empirical formula^a^	H_540_Na_14_K_5_LaP_2_W_34_O_388_	H_334_Na_13_K_6_CeP_2_W_34_O_285_	H_340_Na_12_K_7_EuP_2_W_34_O_288_	H_376_Na_12_K_7_GdP_2_W_34_O_306_	H_400_Na_15_K_4_YbP_2_W_34_O_318_	H_400_Na_14_K_5_LuP_2_W_34_O_318_	H_570_Na_16_K_3_YP_2_W_34_O_403_
fw,^a^ g mol^–1^	13721.40	11883.08	11965.08	12294.66	12478.31	12496.35	13909.42
cryst syst	triclinic	triclinic	triclinic	triclinic	triclinic	triclinic	triclinic
space group	*P*-1	*P*-1	*P*-1	*P*-1	*P*-1	*P*-1	*P*-1
a, Å	14.6480 (1)	14.6177 (1)	14.6273 (1)	14.6430 (1)	14.0833 (1)	14.6306 (1)	14.0617 (1)
b, Å	24.6416 (2)	24.5899 (2)	24.5439 (2)	24.5399 (2)	24.5890 (2)	24.5442 (2)	24.5663 (1)
c, Å	26.2230 (2)	26.1781 (2)	26.1355 (2)	26.1331 (2)	25.1215 (1)	26.1371 (2)	25.1754 (1)
α, deg	65.852 (1)	65.813 (1)	65.657 (1)	65.598 (1)	90.020 (1)	65.312 (1)	90.08 (1)
β, deg	88.193 (1)	88.221 (1)	88.268 (1)	88.218 (1)	102.067 (1)	88.008 (1)	102.00 (1)
γ, deg	74.770 (1)	74.820 (1)	75.014 (1)	75.031 (1)	92.222 (1)	75.000 (1)	92.28 (1)
volume, Å^3^	8302.53 (13)	8253.00 (13)	8226.84 (13)	8230.97 (13)	8500.49 (10)	8208.45 (13)	8499.52 (8)
Z	2	2	2	2	2	2	2
*D*_calc_, g cm^–3^	5.489	4.782	4.830	4.961	4.875	5.056	5.435
abs coeff, mm^–1^	24.17	24.27	24.48	24.50	23.83	24.73	23.67
*F*(000)	12992	10950	11036	11398	11602	11620	13224
⊖ range data collection, °	2.3–34.3	2.3–34.0	2.3–34.0	2.3–34.0	2.5–34.0	2.5–34.1	2.3–34.0
completeness to Θ_max, %_	99.7	99.8	99.7	99.7	99.8	99.7	99.8
index ranges	–22 ≤ *h* ≤ 23, –38 ≤ *k* ≤ 38, –41 ≤ *l* ≤ 40	–22 ≤ *h* ≤ 22, –37 ≤ *k* ≤ 38, –39 ≤ *l* ≤ 40	–22 ≤ *h* ≤ 22, –37 ≤ *k* ≤ 38, –39 ≤ *l* ≤ 40	–22 ≤ *h* ≤ 22, –37 ≤ *k* ≤ 37, –39 ≤ *l* ≤ 38	–20 ≤ *h* ≤ 21, –38 ≤ *k* ≤ 37, –39 ≤ *l* ≤ 39	–22 ≤ *h* ≤ 22, –38 ≤ *k* ≤ 37, –40 ≤ *l* ≤ 38	–21 ≤ *h* ≤ 19, –37 ≤ *k* ≤ 38, –38 ≤ *l* ≤ 39
reflns collected	542856	532967	530334	534318	543992	525844	560601
unique reflns	61820	61824	61272	61306	63082	60887	63560
*R*(int)	0.134	0.256	0.303	0.168	0.330	0.156	0.108
data/restraints/parameter	61820/0/1167	61824/0/1184	61272/0/1092	61306/0/1193	63082/0/1118	60887/0/1164	63560/0/1181
GOF on *F* ^2^	1.002	1.003	1.001	1.002	1.004	1.004	1.004
*R*_1_ ^b^[*I* > 2σ(*I*)]	0.057	0.077	0.097	0.057	0.096	0.064	0.042
*wR*_2_ ^c^ (all data)	0.166	0.209	0.270	0.159	0.258	0.192	0.114

a The formulas and molar masses are based on elemental analysis representing true bulk composition.

b R_1_ = Σ||*F*_*o*_| – |*F*_*c*_||/Σ|F_o_|.

c *w*R_2_ = [Σ*w*(*F*_*o*_^2^ – *F*_*c*_^2^)^2^/Σ*w*(*F*_*o*_^2^)^2^]^1/2^.

Photophysical studies of the Eu^3+^- and Yb^3+^-containing polyanions were performed using a Horiba quantum master spectrometer with a R920 photomultiplier tube detector. The powdered samples were placed in between two quartz plates with a drop of perfluorinated oil and mounted on a Sumitomo closed-cycle He-cryostat for temperature-dependent measurements. A 400 nm long pass filter was used to cut out the second- and higher-order diffraction peaks in the spectra. Correction files supplied by the manufacturer were used to obtain the corrected emission spectra. The data plotting and decay fitting were performed using Origin 24.

#### Synthesis of K_7_[H_4_PW_18_O_62_]·18H_2_O (K-PW_18_)

Following the literature procedure,^7d^ Na_2_WO_4_·2H_2_O (240 g, 0.73 mol) was dissolved in 300 mL of water. The solution was acidified by 160 mL of 4 M HCl under vigorous stirring. Then, a mixture of 4 M HCl and 1 M H_3_PO_4_ was added. The pH was adjusted to 2 and then the solution was refluxed at 120 °C for 96 h. After cooling, the solution was treated with 100 g KCl. The precipitate was redissolved in 150 mL H_2_O and heated at 80 °C for 48 h. After cooling, the clear solution was treated with 30 g KCl to give a fine yellow powder (yield 140 g, 58%).

#### Synthesis of K_11_[H_4_PW_17_O_61_]·18H_2_O (K-PW_17_)

Following the literature procedure,^7d^ a sample of K_7_[H_4_PW_18_O_62_]·18H_2_O (8.00 g, 1.62 mmol) was dissolved in 20 mL of water while stirring. To the clear solution, 17 mL of 1 M KHCO_3_ was added, and then a white precipitate formed. Stirring continued for roughly one more hour. The solid was left to settle and was filtered, washed twice with ethanol, twice with diethyl ether, and then air-dried (yield 6.5 g, 81%).

#### Synthesis of Na_14_K_5_[La­(P­(H_4_)­W_17_O_61_)_2_]·266H_2_O (NaK–LaPW_17_)

LaCl_3_·7H_2_O (0.027 g, 0.11 mmol) was dissolved in 20 mL of a 1 M sodium acetate at pH adjusted to 6.0, and then K_11_[H_4_PW_17_O_62_]·18H_2_O (0.50 g, 0.11 mmol) was added in small portions while stirring at room temperature. After an hour, the mixture was filtered, and the filtrate was left to crystallize at room temperature in an open vial. After a week, colorless block-shaped crystals of **NaK–LaPW**_**17**_ were collected and left to dry in air. Yield 0.29 g (33.9% based on the limiting reagent **K-PW**_**17**_); Anal. Calcd (%): Na 2.35, K 1.52, P 0.53, W 45.56, La 1.01; found Na 2.42, K 1.91, P 0.61, W 45.50, La 1.08. IR absorption bands (cm^–1^): 1037 (s), 976 (sh), 932 (s), 845 (w), 757 (m), 767 (m) 593 (w), 513 (m).

#### Synthesis of Na_13_K_6_[Ce­(P­(H_4_)­W_17_O_61_)_2_]·163H_2_O (NaK–CePW_17_)

The same synthetic procedure was followed, but using CeCl_3_·7H_2_O (0.041 g, 0.11 mmol) instead of LaCl_3_·7H_2_O. Yield 0.21 g (20.6% based on the limiting reagent **K-PW**_**17**_); Anal. Calcd (%): Na 2.52, K 1.97, P 0.52, W 52.60, Ce 1.18; found Na 2.74, K 2.01, P 0.62, W 52.60, Ce 1.43. IR absorption bands (cm^–1^): 1037 (s), 976 (sh), 932 (s), 845 (w), 757 (m), 767 (m) 593 (w), 513 (m).

#### Synthesis of Na_12_K_7_[Eu­(P­(H_4_)­W_17_O_61_)_2_]·166H_2_O (NaK-EuPW_17_)

The same synthetic procedure was followed using EuCl_3_·6H_2_O (0.040 g, 0.11 mmol). Yield 0.20 g (19.6% based on the limiting reagent **K-PW**_**17**_); Anal. Calcd (%): Na 2.31, K 2.29, P 0.52, W 52.24, Eu 1.27; found Na 2.55, K 2.37, P 0.62, W 52.50, Eu 1.31. IR absorption bands (cm^–1^): 1037 (s), 976 (sh), 932 (s), 845 (w), 757 (m), 767 (m) 593 (w), 513 (m).

#### Synthesis of Na_12_K_7_[Gd­(P­(H_4_)­W_17_O_61_)_2_]·184H_2_O (NaK-GdPW_17_)

The same synthetic procedure was followed using GdCl_3_·6H_2_O (0.040 g, 0.11 mmol. Yield 0.23 g (22.5% based on the limiting reagent **K-PW**_**17**_); Anal. Calcd (%): Na 2.42, K 2.23, P 0.52, W 50.91, Gd 1.28; found Na 2.55, K 2.31, P 0.70, W 50.90, Gd 1.30. IR absorption bands (cm^–1^): 1037 (s), 976 (sh), 932 (s), 845 (w), 757 (m), 767 (m) 593 (w), 513 (m).

#### Synthesis of Na_15_K_4_[Yb­(P­(H_4_)­W_17_O_61_)_2_]·196H_2_O (NaK-YbPW_17_)

The same synthetic procedure was followed using Yb­(NO_3_)_3_·6H_2_O (0.042 g, 0.11 mmol). Yield 0.22 g (20.9% based on the limiting reagent **K-PW**_**17**_); Anal. Calcd (%): Na 3.76, K 1.25, P 0.50, W 50.09, Yb 1.31; found Na 4.13, K 1.19, P 0.53, W 50.10, Yb 1.26. IR absorption bands (cm^–1^): 1037 (s), 976 (sh), 932 (s), 845 (w), 757 (m), 767 (m) 593 (w), 513 (m).

#### Synthesis of Na_14_K_5_[Lu­(P­(H_4_)­W_17_O_61_)_2_]·130H_2_O (NaK-LuPW_17_)

The same synthetic procedure was followed using Lu­(NO_3_)_3_·6H_2_O (0.039 g, 0.11 mmol). Yield 0.19 g (19.4% based on the limiting reagent **K-PW**_**17**_); Anal. Calcd (%): Na 2.85, K 1.73, P 0.55, W 55.29, Lu 1.61; found Na 3.14, K 1.85, P 0.69, W 55.30, Lu 1.70. IR absorption bands (cm^–1^): 1037 (s), 976 (sh), 932 (s), 845 (w), 757 (m), 767 (m) 593 (w), 513 (m).

#### Synthesis of Na_16_K_3_[Y­(P­(H_4_)­W_17_O_61_)_2_]·281H_2_O (NaK-YPW_17_)

The same synthetic procedure was followed using YCl_3_·6H_2_O (0.033 g, 0.11 mmol). Yield 0.21 g (23.4% based on the limiting reagent **K-PW**_**17**_); Anal. Calcd (%): Na 2.64, K 0.84, P 0.45, W 44.94, Y 0.64; found Na 2.92, K 2.37, P 0.65, W 44.80, Y 0.65. IR absorption bands (cm^–1^): 1037 (s), 976 (sh), 932 (s), 845 (w), 757 (m), 767 (m) 593 (w), 513 (m).

## Results and Discussion

### Synthesis and Structure

We succeeded in synthesizing a family of lanthanide-containing 17-tungsto-1-phosphates of the general formula [Ln­(P­(H_4_)­W_17_O_61_)_2_]^19–^ (**LnPW**_**17**_; Ln = La^3+^, Ce^3+^, Eu^3+^, Gd^3+^, Yb^3+^, Lu^3+^, Y^3+^), which crystallized as hydrated mixed sodium–potassium salts in the triclinic space group P1̅. All polyanions exhibit an idealized *C*_2_ symmetry in the solid state, comprising two monovacant, asymmetric Wells-Dawson units [(H_4_)­PW_17_O_61_]^11−^ being coordinated to the lanthanide ion in a *syn*-configuration, with the phosphate heterogroup residing next to the lacunary site (Figure 1).

**Figure 1:**
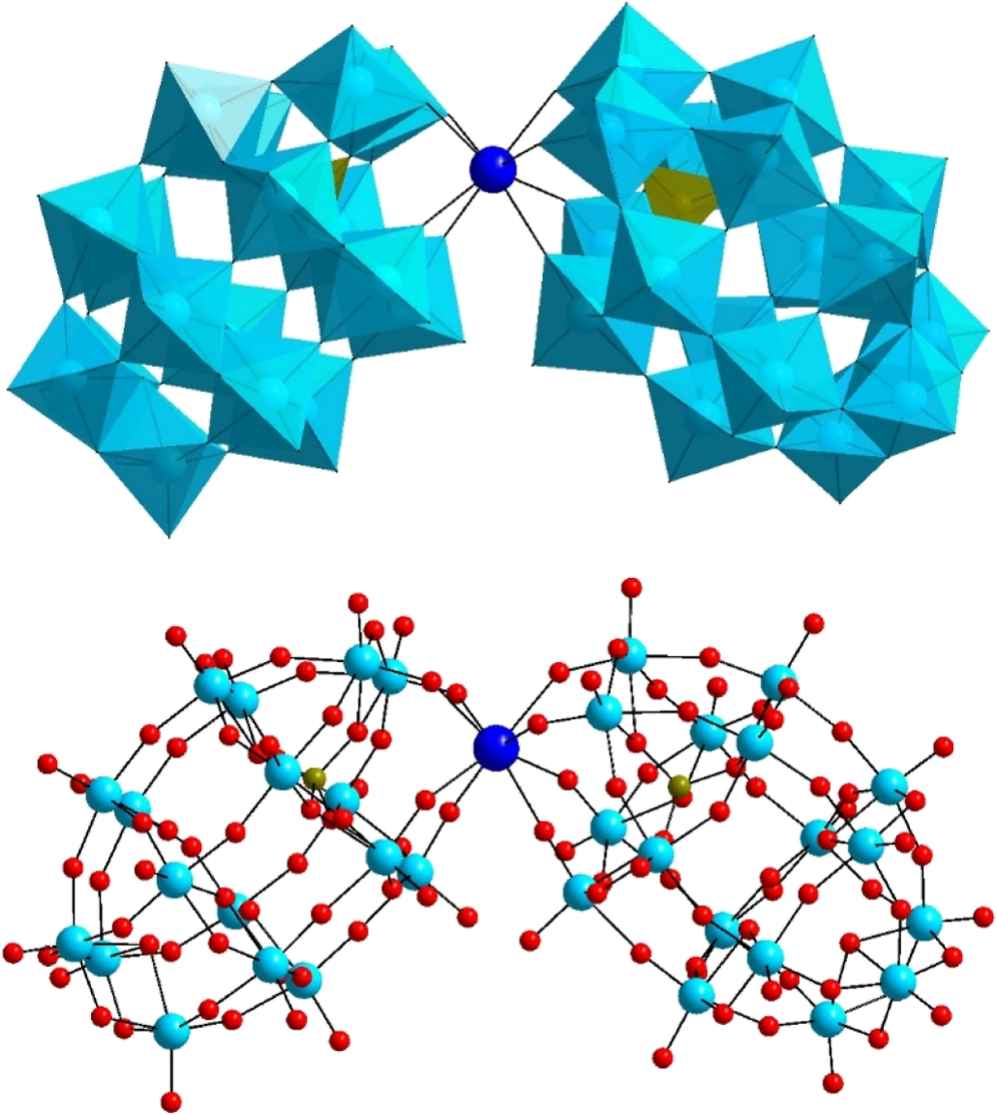
Polyhedral (top) and ball-and-stick (bottom) representation of the polyanion family [Ln­(P­(H_4_)­W_17_O_61_)_2_]^19–^ (**LnPW**_**17**_; Ln = La^3+^, Ce^3+^, Eu^3+^, Gd^3+^, Yb^3+^, Lu^3+^, Y^3+^). Color code: WO_6_ octahedra (sky blue), PO_4_ tetrahedra (dark yellow), oxygen (red), lanthanide (blue), tungsten (dark green), and phosphorus (dark yellow).

It should be emphasized that two asymmetric Wells-Dawson ions are present in these polyanions, which have only one P heteroatom each, whereas the other lacunary site is occupied by four protons. The Ln^3+^ center adopts square-antiprismatic coordination geometry, being coordinated to four oxo-donors from each asymmetric Wells-Dawson unit.^2^ The isostructural series **LnPW**_**17**_ differs mainly in terms of the average Ln–O bond lengths. As can be seen in the graph of (Figure 2), the expected correlation exists between the ionic radii of the lanthanide ions and the average Ln–O bond lengths, namely that the latter decreases with decreasing size of the lanthanide ion.^9a^ Careful tuning of the reaction conditions, in particular solution pH, reaction temperature, and solvent medium, was crucial to identify the optimal synthetic conditions for **LnPW**_**17**_.

**Figure 2:**
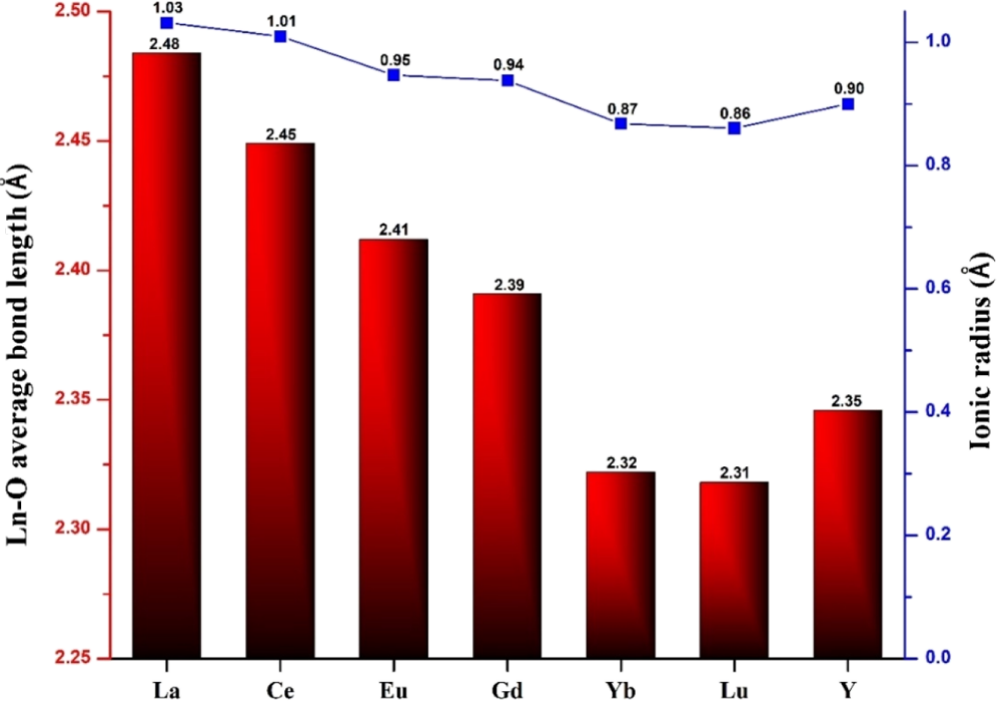
Lanthanide ionic radii (blue squares) and Ln–O bond lengths (red bars) for [Ln­(P­(H_4_)­W_17_O_61_)_2_]^19–^ (**LnPW**_**17**_; Ln = La^3+^, Ce^3+^, Eu^3+^, Gd^3+^, Yb^3+^, Lu^3+^, Y^3+^).

FT-IR spectroscopy was used to obtain information on the vibrational modes of the title polyanions. The monovacant POM precursor salt K_11_[P­(H_4_)­W_17_O_62_] exhibits characteristic P–O stretching vibrations at 1066 and 1032 cm^–1^ (Figure S1). For the series of our [Ln­(P­(H_4_)­W_17_O_61_)_2_]^19–^ (**LnPW**_**17**_; Ln = La^3+^, Ce^3+^, Eu^3+^, Gd^3+^, Yb^3+^, Lu^3+^) polyanion salts, only a single band at ∼ 1033 cm^–1^ is observed, a diagnostic change indicative of symmetry lowering resulting from lanthanide coordination at the vacant site. Further vibrational assignments include the terminal W=O stretching bands at 870 and 720 cm^–1^, consistent with the preservation of the tungsten-oxo framework. Broad absorptions from 3000 to 3600 cm^–1^ and a band at 1625 cm^–1^ correspond to O–H stretching and bending modes of coordinated and lattice water, respectively. The nearly identical spectral features across the polyanion series **LnPW**_**17**_ confirm the isostructural nature of the title compounds.

The seven polyanions **LnPW**_**17**_ are formed in aqueous medium at pH 6.0, essentially independent of the reaction temperature. The optimized reaction conditions, which led to the highest crystalline yield, were found to be at room temperature in 1 M sodium acetate buffer at pH 6.0, with yields in the range of 20–35%. All our compounds are novel, except the cerium derivative, which was reported by Pope and co-workers.^7e^ However, our synthetic conditions are not only different but also easier, as we employ one-pot open-beaker conditions rather than a multistep approach using large quantities and hydrothermal heating.^7e^

The phase purity of the crystalline bulk materials was confirmed by powder X-ray diffraction (PXRD). The experimental PXRD patterns for all seven **NaK-LnPW**_**17**_ compounds are in good agreement with the simulated patterns derived from the single-crystal X-ray data (Figure S2), confirming the bulk purity of the samples.

Thermogravimetric analysis (TGA) was carried out for the seven compounds **NaK-LnPW**_**17**_ (Ln = La^3+^, Ce^3+^, Eu^3+^, Gd^3+^, Yb^3+^, Lu^3+^, Y^3+^) in the temperature range of 25–600 °C under N_2_ atmosphere to evaluate their thermal stability. The thermograms of all compounds exhibited nearly identical behavior, showing a gradual weight loss primarily attributed to the release of lattice and coordinated water molecules. No significant decomposition of the polyoxometalate framework was observed within the studied temperature range, indicating that all compounds possess high thermal stability (Figure S3).

#### NMR Spectroscopy

^31^P NMR spectroscopy was employed to probe the stability of the title polyanions in solution. For the reference compounds, such as the asymmetric plenary Wells-Dawson ion [P­(H_4_)­W_18_O_62_]^7–^ and the monovacant derivative [P­(H_4_)­W_17_O_61_]^11–^ species show sharp singlets at −7.1 and −6.8 ppm, respectively (Figure S4). The ^31^P NMR spectra of [Ln­(P­(H_4_)­W_17_O_61_)_2_]^19–^ (**LnPW**_**17**_; Ln = La^3+^, Ce^3+^, Eu^3+^, Yb^3+^, Lu^3+^, Y^3+^) exhibit single ^31^P resonances (Figure 3), confirming the structural stability of the polyanions in solution. As expected, the chemical shifts vary quite a bit across the polyanion series as a function of the electronic and magnetic properties of the incorporated Ln^3+^ ion (δ = −2.9, **LaPW**_**17**_; −5.6, **CePW**_**17**_; 7.4, **EuPW**_**17**_; 37.4, **YbPW**_**17**_; −3.3, **LuPW**_**17**_; −3.3, **YPW**_**17**_ (Figure 3). The observed downfield shift in the case of **YbPW**_**17**_ and **EuPW**_**17**_ arises from paramagnetic deshielding effects, consistent with known trends in POM-lanthanide NMR spectroscopy. The absence of any ^31^P signal in the **GdPW**_**17**_ derivative reflects significant line broadening caused by the highly paramagnetic Gd^3+^ ion (seven unpaired 4f electrons), which enhances spin–lattice relaxation and renders the signal unobservable. Notably, time-dependent ^31^P NMR monitoring of the lanthanum derivative **LaPW**_**17**_ in 0.5 M sodium acetate solution at pH 6.0 showed no change over 6 weeks, establishing the long-term solution-phase stability of the polyanions (Figure S5).

**Figure 3:**
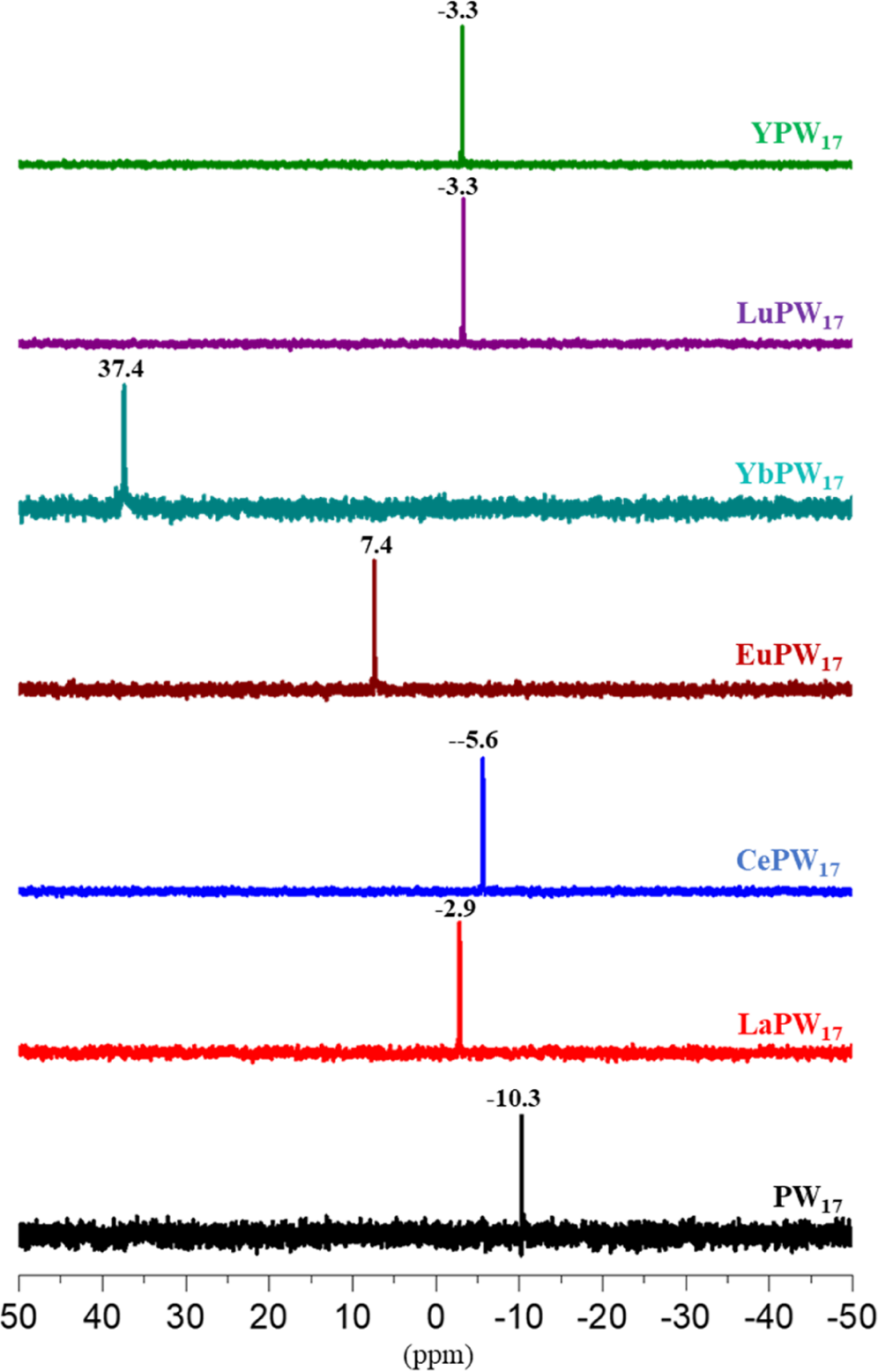
Room temperature ^31^P NMR spectra of the title polyanions [Ln­(P­(H_4_)­W_17_O_61_)_2_]^19–^ (**LnPW**_**17**_; Ln = La^3+^, Ce^3+^, Eu^3+^, Yb^3+^, Lu^3+^, Y^3+^) and the [P­(H_4_)­W_17_O_62_]^11–^ reference ion, all dissolved in 0.5 M CH_3_COONa at pH 6.0.

^183^W NMR spectroscopy on **LaPW**_**17**_ further confirmed the structural integrity of the polyanion in solution. The spectrum revealed eight distinct resonances at δ = −132, −159, −173, −178, −190, −205, −211, and −221 ppm (Figure S6), corresponding to the eight pairs of structurally inequivalent W sites within each {P­(H_4_)­W_17_} unit of the polyanion, consistent with the solid-state structure. We cannot identify with confidence the ninth signal with half intensity, corresponding to the unique tungsten atom in the cap opposite the lacunary site, due to the low intensity. Nevertheless, the identity of the polyanion is established.

### Magnetic Measurements

#### DC Studies

The magnetic properties of the **NaK**-**CePW**_**17**_, **NaK**-**GdPW**_**17,**_ and **NaK**-**YbPW**_**17**_ polyanion salts were investigated through magnetic susceptibility measurements employing a SQUID magnetometer. The static magnetic susceptibility χ_M_ *T*(*T*) data for powdered samples in an applied field of 1 kOe, 5 kOe and 10 kOe. For **NaK**-**CePW**_**17**_ and **NaK**-**YbPW**_**17**_ the data is rather steep and discontinuous, probably a direct consequence of the low magnetic moment associated with these lanthanide ions (the theoretically expected room-temperature χ_M_ *T* value would be 0.8 cm^3^ mol^–1^ K for Ce^3+^ (*S* = 1/2, *L* = 3, *J* = 5/2 and *g*_*J*_ = 6/7), and 2.6 cm^3^ mol^–1^ K for Yb^3+^ (*S* = 1/2, *L* = 3, *J* = 7/2 and *g*_*J*_ = 8/7)) and the low metal mass percent in the sample (1.5% for Ce^3+^ and 1.9% for Yb^3+^), Figure S7. Due to these characteristics, it is not possible to obtain a meaningful χ_M_ *T*(*T*) for these systems; however, based on the single-ion and quasi-isolated (magnetically) nature of the Ln^3+^ ions, a negligible interaction is expected for these systems.

Despite the low (1.8%) lanthanide content for **NaK**-**GdPW**_**17**_, the magnetic moment for Gd^3+^ is stronger than for Ce^3+^ and Yb^3+^; hence, it was possible to obtain a representative χ_M_ *T*(*T*) and *M*(*H*) profile. The room temperature χ_M_ *T* value was found to be 7.9 cm^3^ mol^–1^ K in agreement with an isolated Gd^3+^ ion (7.87 cm^3^ mol^–1^ K for Gd^3+^ with *S* = 7/2, and *g* = 2.0), (Figure 4A). Upon cooling, the χ_M_ *T* product remained practically constant down to 2 K, implying negligible/no interactions. The *M*(*H*) measurements between 0 and 7 T and 2 and 6 K revealed a saturation value of 7 *N*_A_μ_B_ as expected for the Gd^3+^ ion, reaching saturation above 3 T (Figure 4B). A comparison of the *M*(*H*) traces with the Brillouin function is almost superimposable, confirming the system to be a *S* = 7/2 state.

**Figure 4:**
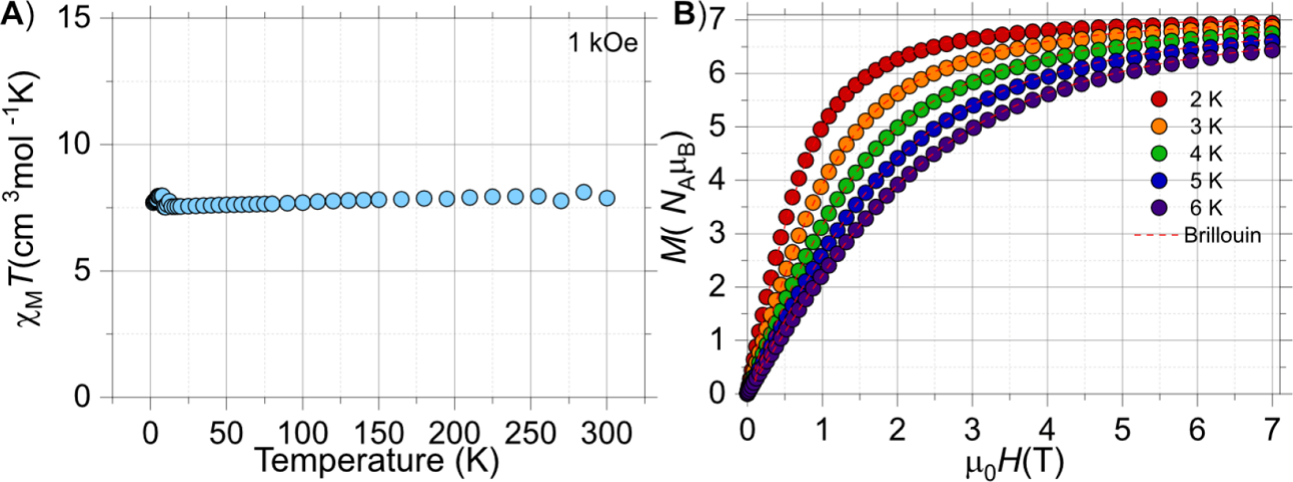
Experimental χ_M_ *T*(*T*) for (A) **NaK**-**GdPW**_**17**_ and (B) *M*(*H*) data collected at different temperatures. The solid lines in (B) are the Brillouin function for *S* = 7/2 and *g* = 2.0.

#### AC Studies

Despite the low magnetic moment exhibited by **NaK**-**CePW**_**17**_ and **NaK**-**YbPW**_**17**_, these samples are excellent test subjects to prove the so-called single-molecule magnet (SMM) behavior. A frequency and temperature-dependent behavior was observed for both samples under applied fields. For **NaK**-**CePW**_**17**_, an applied field of 1.3 kOe was employed for data collection, while the AC field was 6 Oe (Figure 5). As expected, a rather small signal is obtained for the system; however, clearly revealing a maximum in the out-of-phase (χ”_M_) centered at 140 Hz at 2 K (Figure 5B). This maximum shift toward higher frequencies upon temperature increments up to 4 K, where the maximum is out of the operational frequency window of the instrument. Simultaneously fitting the in-phase (χ′_M_) and χ″_M_ to a generalized Debye model allows the extraction of the temperature relaxation times (τ­(*T*)) (Figure 5D).

**Figure 5:**
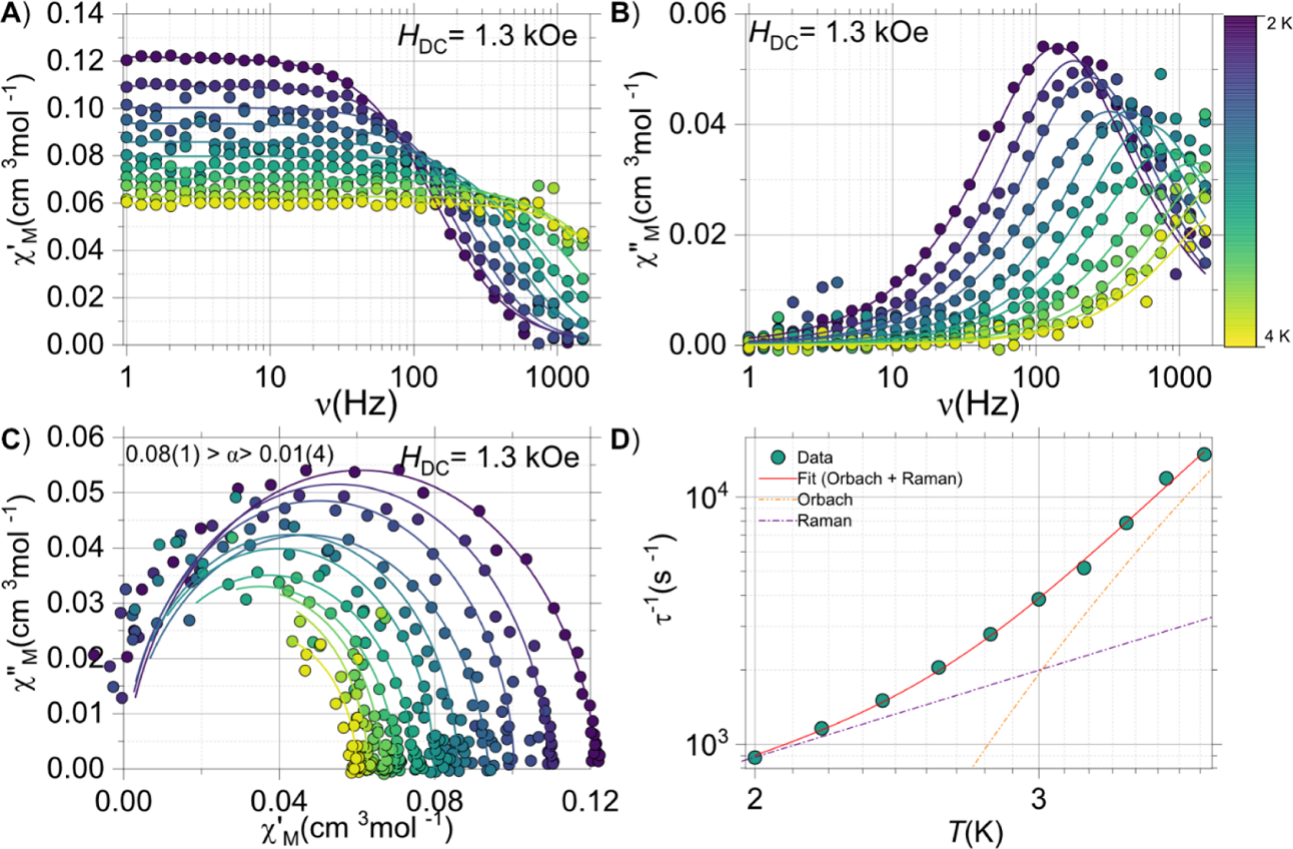
AC data for **NaK**-**CePW**_**17**_: (A) χ′_M_(ν;*T*); (B) χ′_M_(ν;*T*); (C) χ′_M_ vs χ′_M_ (Cole–Cole plot) and (D) experimental τ­(*T*) data, fit (solid line) and decomposition of the processes contributing to the overall fit (dashed lines). The solid lines in panels A-C fit a generalized Debye model.

For **NaK**-**YbPW**_**17**_, a DC field of 1.9 kOe was employed for the full data collection. The maximum for this system occurs between 2 and 6.8 K. The maximum of 16 Hz is observed at the lowest temperature, which shifts swiftly toward higher frequencies upon temperature increment (Figure 6B). Likewise, simultaneous fitting of the χ′_M_(ν;*T*) and χ″_M_(ν;*T*) to a generalized Debye analysis also yields the τ­(*T*) data (Figure 6D).

**Figure 6:**
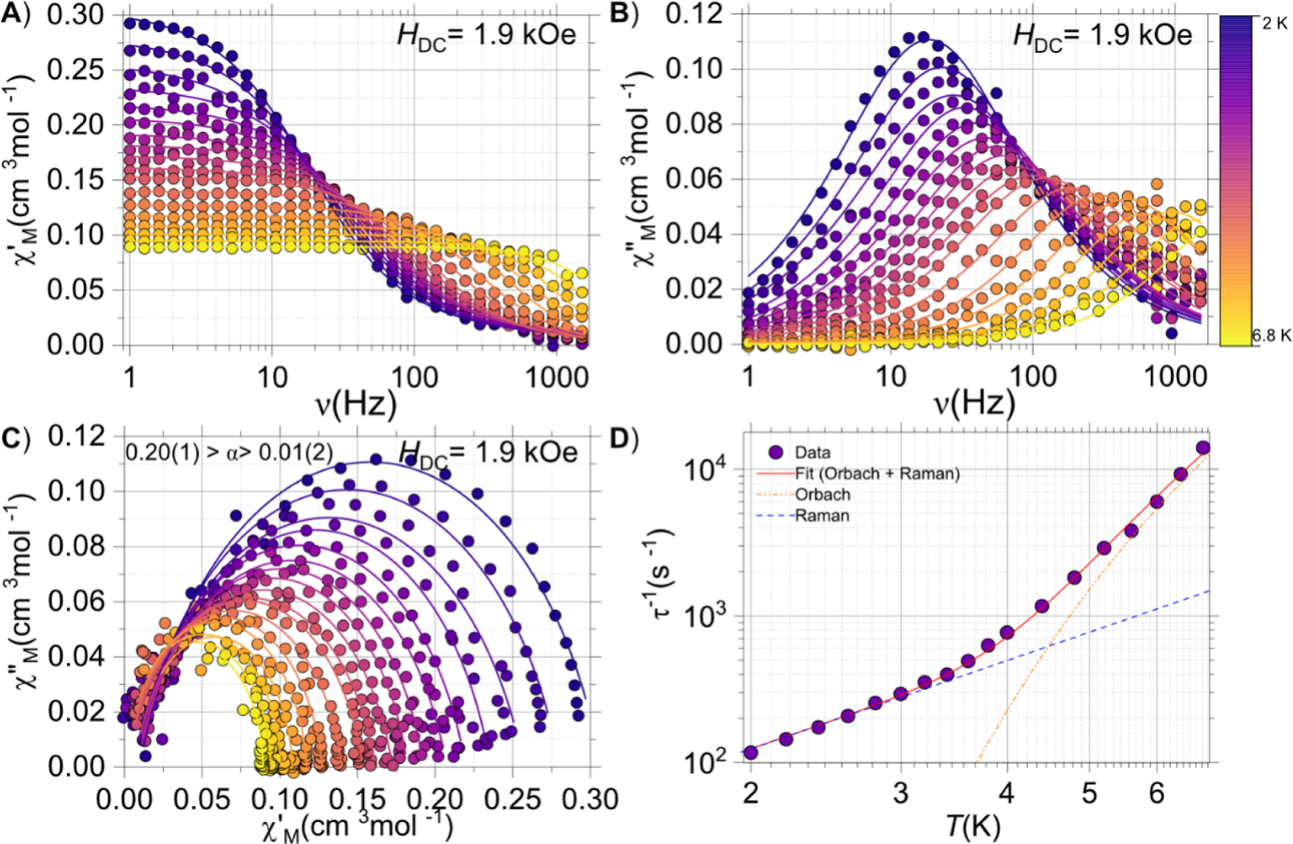
AC data for **NaK**-**YbPW**_**17**_: (A) χ′_M_(ν;*T*); (B) χ′_M_(ν;*T*); (C) χ′_M_ vs χ′_M_ (Cole–Cole plot) and (D) experimental τ­(*T*) data, fit (solid line) and decomposition of the processes contributing to the overall fit (dashed lines). The solid lines in panels A-C fit a generalized Debye model.

To gain further insight into the relaxation characteristics of the system, the τ­(*T*) data can be fitted to (1)

$$\tau^{-1}=\tau_{0}^{-1}\exp\left(-\frac{U_{eff}}{k_{B}T}\right)+C\frac{\exp\left(\hbar \omega /k_{B}T\right)}{{\left(\exp\left(\frac{\hbar \omega }{k_{B}T}\right)-1\right)}^{2}}$$ 1

where the first term is the Orbach process and the second represents the vibrational-dependent Raman term, ℏω, the vibrational mode contributing to this mechanism. For **NaK**-**CePW**_**17**_, the fits yield the following parameters: *U*_eff_ = 18(1) cm^–1^, τ_0_ = 8(3)×10^–8^ s, *C* = 0.001(20) s^–1^ and ℏω = 0.002(10) cm^–1^. For **NaK**-**YbPW**_**17**_, the following values were obtained: *U*_eff_ = 26(1) cm^–1^, τ_0_ = 3.2(6)×10^–7^ s, *C* = 0.02(70) s^–1^ and ℏω = 0.02(30) cm^–1^. Keeping in mind that we consider the vibrational Raman mechanism, it is expected that the barriers are close to the actual ground-to-first excited state separation. Unfortunately, the large size of the polyanions precludes the investigation, at this stage, of the energy manifold of these systems via Complete Active Space Self-consistent field (CASSCF) calculations.

#### μSQUID Investigations

Sub-kelvin studies of the three samples **NaK**-**CePW**_**17**_, **NaK**-**GdPW**_**17**_, and **NaK**-**YbPW**_**17**_ were carried out employing μSQUID arrays. In all cases, the field was aligned along the easy axis of the crystal employing the transverse field method.^11a^ For **NaK**-**CePW**_**17**_ the loops are close at zero fields, while some openings are present at larger fields. The closed-loop behavior at zero field is consistent with fast quantum tunnelling of the magnetization (QTM) relaxation process (Figure 7A,B). At higher fields, the loops are open, signaling phonon bottleneck effects.^11b^ Above 200 mK, the magnetic signal decreases drastically. For **NaK**-**GdPW**_**17**_, the loops are closed at all temperatures and sweep rates, indicating very fast relaxation. Furthermore, a small interaction can be visible at low fields, which could account for intermolecular dipolar interactions (Figure 7C,D). For **NaK**-**YbPW**_**17**_, at 30 mK the loops are slightly open at nonzero field and fast sweep rates, while at zero field, a sharp step indicates fast QTM (Figure 7E,F). For **NaK**-**CePW**_**17**_ and **NaK**-**YbPW**_**17**_ the open loops demonstrate some anisotropy of these systems, in line with the AC studies, where frequency-dependent behavior was observed when a DC field was applied. Hence, we find **NaK**-**CePW**_**17**_ and **NaK**-**YbPW**_**17**_ to be field-induced SMMs. The anisotropy of these systems is expected to arise from the ligand field and geometry of the lanthanide when embedded in the POM.^12,13^

**Figure 7:**
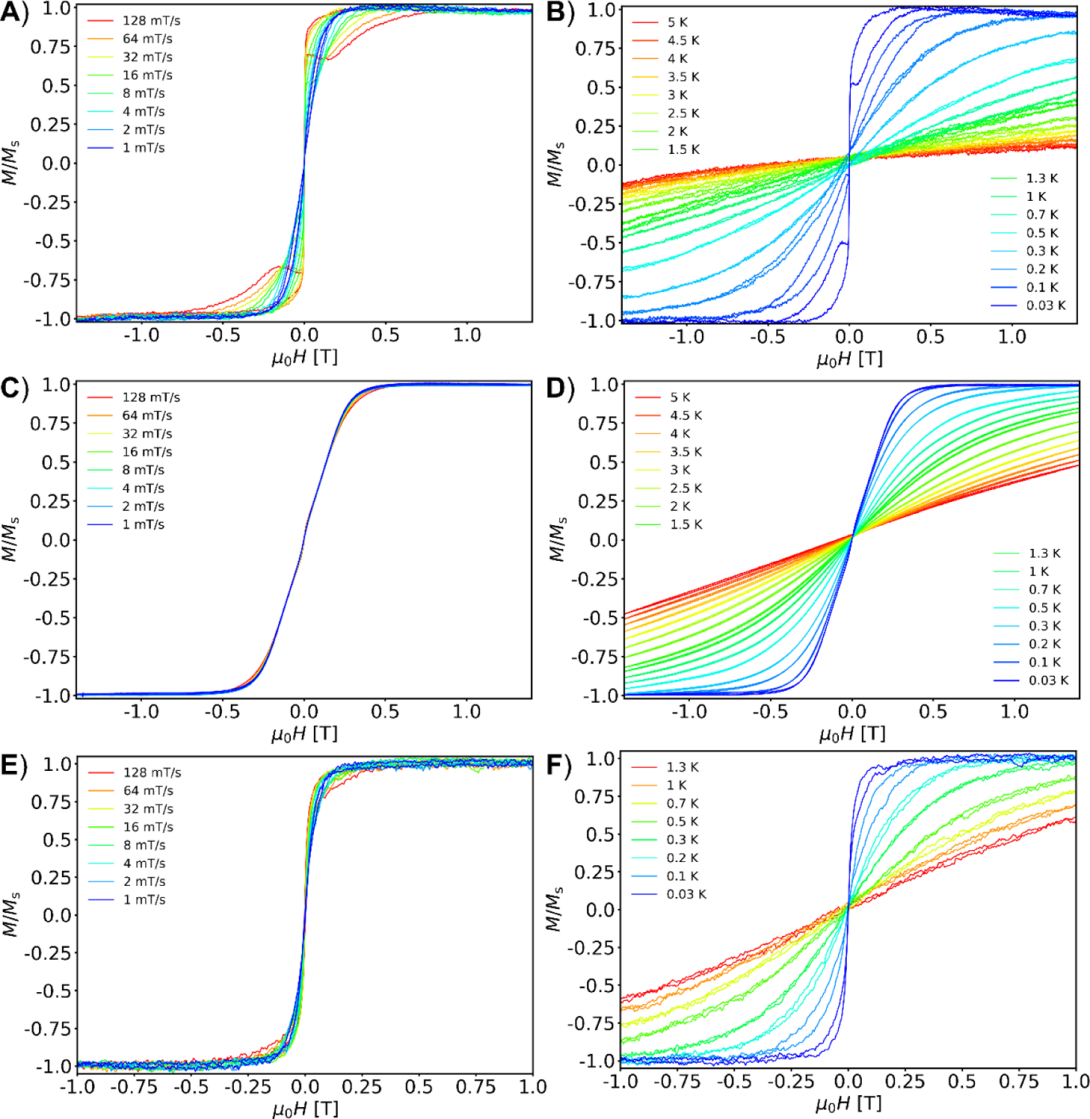
Sweep rate-dependent μSQUID studies at a fixed temperature of 30 mK and temperature-dependent at a fixed sweep rate of 16 mT/s for **NaK**-**CePW**_**17**_ (A,B), **NaK**-**GdPW**_**17**_ (C,D), and **NaK**-**YbPW**_**17**_ (E,F), respectively.

#### Photoluminescence Studies

Temperature-dependent emission studies on **NaK-EuPW**_**17**_ revealed the characteristic ^5^D_0_ → ^7^F_*J*_ (*J* = 0–4) transitions upon excitation of the Eu^3+^-based ^7^F_0_ → ^5^L_6_ transition at 395 nm, as shown in (Figure 8). Apart from the ^5^D_0_ → ^7^F_*J*_ (*J* = 0–4) transitions, we have also observed the ^5^D_1_ → ^7^F_*J*_ (*J* = 0–2), ^5^D_2_ → ^7^F_*J*_ (*J* = 0, 2, or 3), and ^5^D_3_ → ^7^F_*J*_ (*J* = 2–3) transitions (Figure 9 and Table 2) involving the ^5^D_1_, ^5^D_2,_ and ^5^D_3_ excited states, respectively. The present transitions from the excited ^5^D_*J*_ manifold indicate the absence of ^5^D_1_ → ^5^D_0_ cross relaxation, a lack of nonradiative relaxation channels in the vicinity of Eu^3+^, and a rigid lattice structure.^13a^ In the excitation spectrum at 2.3 K (Figure 8a), we have observed transitions at 376 nm (^7^F_0_ → ^5^G_4_), 394 nm (^7^F_0_ → ^5^L_6_), 465 nm (^7^F_0_ → ^5^D_2_), 526 nm (^7^F_0_ → ^5^D_1_), and 580 nm (^7^F_0_ → ^5^D_0_). At 300 K, two additional transitions at 416 nm (^7^F_1_ → ^5^D_3_) and 534 nm (^7^F_1_ → ^5^D_1_) are observed (Figure 10), indicating a temperature-induced population of the ^7^F_1_ state. Interestingly, the characteristic O^2–^ → M charge transition involving the tungsten-oxo core of the polyanion is not observed in the excitation spectra at all temperatures.

**Figure 8:**
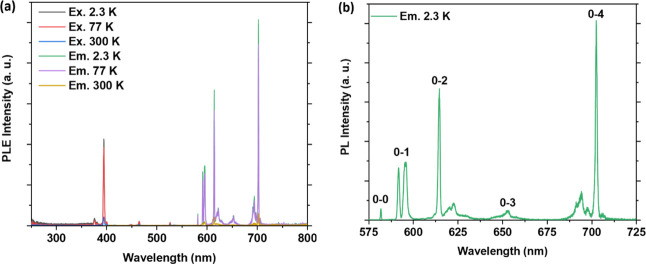
(a) Temperature-dependent photoluminescent excitation (PLE) and emission (PL) spectra of **NaK-EuPW**_**17**_. (b) PL spectrum of the compound at 2.3 K showing the characteristic ^5^D_0_ → ^7^F_*J*_ (*J* = 0–4) transitions. Key: 0–0 (^5^D_0_ → ^7^F_0_), 0–1 (^5^D_0_ → ^7^F_1_), 0–2 (^5^D_0_ → ^7^F_2_), 0–3 (^5^D_0_ → ^7^F_3_), and 0–4 (^5^D_0_ → ^7^F_4_). The compound was excited at 395 nm, and the emission was monitored at 614 nm.

**Figure 9:**
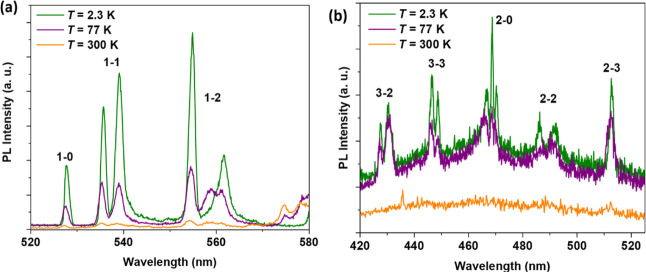
Temperature-dependent PL spectra for **NaK-EuPW**_**17**_ showing (a) ^5^D_1_ → ^7^F_*J*_ (*J* = 0–2) and (b) ^5^D_3_ → ^7^F_*J*_ (*J* = 2–3) and ^5^D_2_ → ^7^F_*J*_ (*J* = 0, 2, or 3) transitions. Key: (a) 1–0 (^5^D_1_ → ^7^F_0_), 1–1 (^5^D_1_ → ^7^F_1_), 1–2 (^5^D_1_ → ^7^F_2_) and (b) 3–2 (^5^D_3_ → ^7^F_2_), 3–3 (^5^D_3_ → ^7^F_3_) and 2–0 (^5^D_2_ → ^7^F_0_); 2–2 (^5^D_2_ → ^7^F_2_), 2–3 (^5^D_2_ → ^7^F_3_).

**Table 2:** Assignment of f–f Transitions Observed for **NaK-EuPW**_**17**_ at 2.3 K^a^

wavelength (nm)	transition
428 to 430	^5^D_3_ → ^7^F_2_
446 to 448	^5^D_3_ → ^7^F_3_
466	^5^D_2_ → ^7^F_0_
486	^5^D_2_ → ^7^F_2_
512	^5^D_2_ → ^7^F_3_
528	^5^D_1_ → ^7^F_0_
534 to 544	^5^D_1_ → ^7^F_1_
552 to 568	^5^D_1_ → ^7^F_2_
581	^5^D_0_ → ^7^F_0_
594	^5^D_0_ → ^7^F_1_
617	^5^D_0_ → ^7^F_2_
652	^5^D_0_ → ^7^F_3_
699	^5^D_0_ → ^7^F_4_

a The transitions from the excited ^5^D_*J*_ manifolds are assigned based on a study by Dejneka et al.^13b^

**Figure 10:**
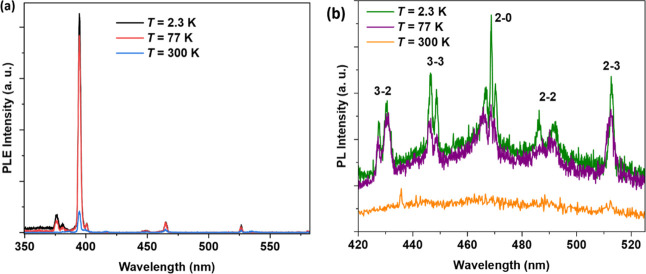
Temperature-dependent PLE spectra for **NaK-EuPW**_**17**_. The emission was monitored at 614 nm. (a) The ^7^F_0_ → ^5^L_6_ transition at 395 nm is the dominant one. (b) Data showing the low-intensity transitions. See text for assignments.

Lifetime studies for **NaK-EuPW**_**17**_ resulted in decay curves that can be satisfactorily fitted with a monoexponential function (Figure 11). At 2.3 K, a ^5^D_0_ lifetime of 2.17 ms is obtained, and a decrease in lifetime—1.84 ms—is noted at 300 K. The CIE (Commission Internationale de l’éclairage) 1931 coordinates obtained for the compound remained almost unchanged in the 2.3 to 300 K range (see Figure 12 and Table 3).

**Figure 11:**
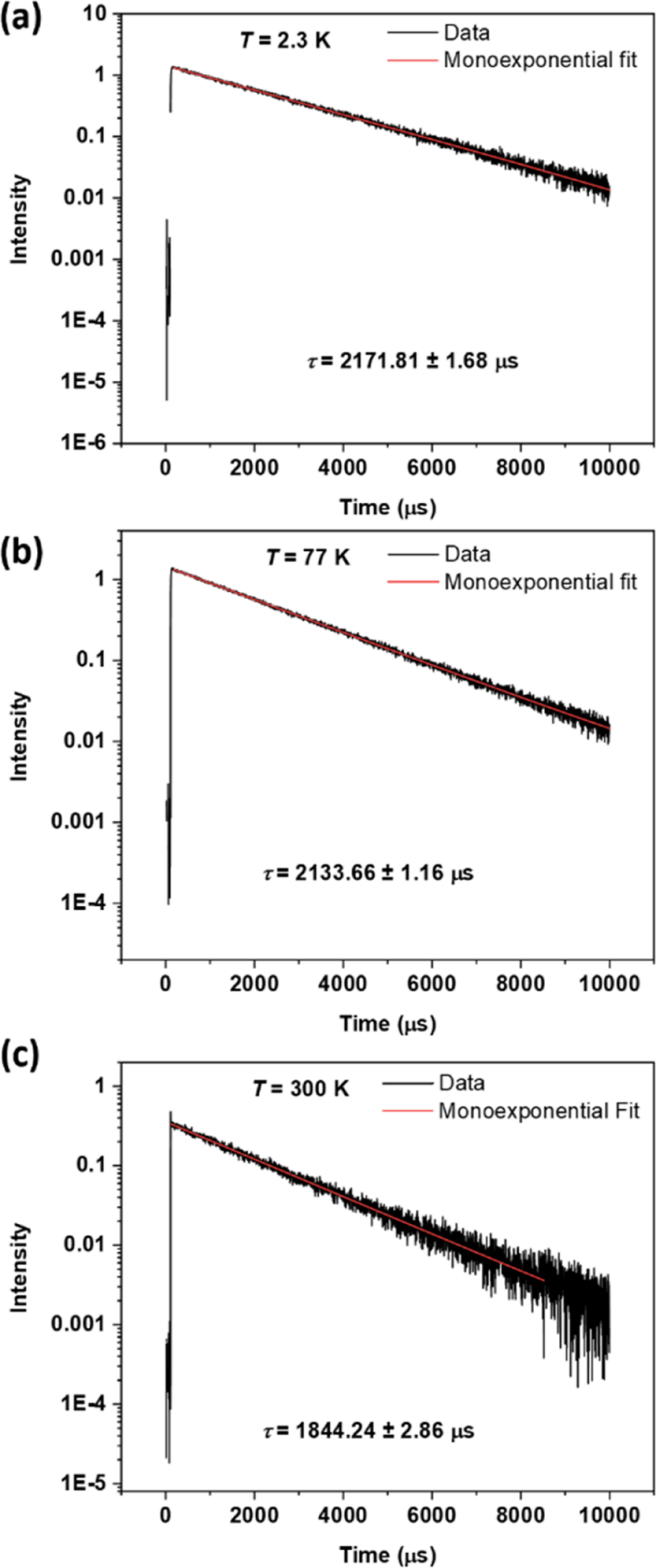
Temperature-dependent PL lifetime studies on **NaK-EuPW**_**17**_ at (a) 2.3 K, (b) 77 K, and (c) 300 K. The compound was excited at 395 nm, and the emission was monitored at 614 nm.

**Figure 12:**
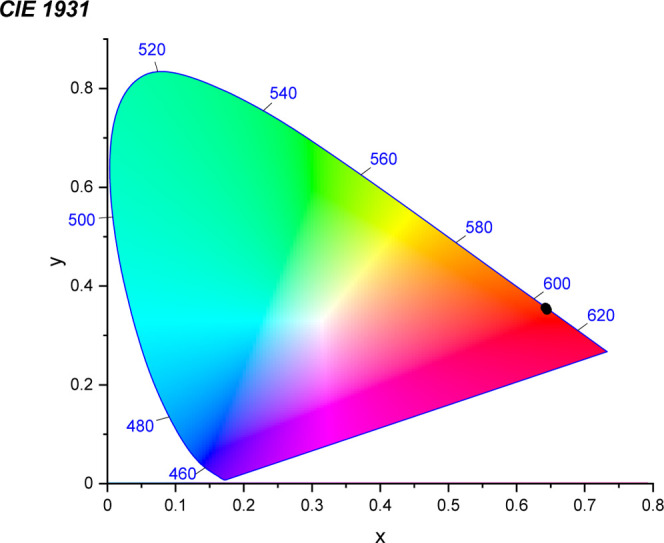
CIE 1931 diagram obtained for **NaK-EuPW**_**17**_.^14^ The black dots indicate the CIE coordinates. Since the variation of the coordinates is minimal with respect to temperature, (see Table 3), all three dots overlap with each other.

**Table 3:** Temperature-dependent CIE Coordinates (*x*, *y*) Obtained for **NaK-EuPW**_**17**_ ^a^

	temperature		
parameter	2.3 K	77 K	300 K
*X*	0.644	0.645	0.646
*Y*	0.355	0.354	0.350

a The compound was excited at 395 nm.

Parameters such as the branching ratio (β_*R*_), asymmetry ratio (R), and Judd-Ofelt (J-O) parameters—Ω_2_, Ω_4_, and Ω_6_—can be obtained from the emission spectra and used to deduce the coordination environment around the Eu^3+^ centers. The branching ratio reflects the intensities of the ^5^D_0_ → ^7^F_*J*_ (*J* = 0, 1, 2, 3, 4) transitions. It is a ratio between the intensity of a particular ^5^D_0_ → ^7^F_*J*_ transition (I­(^5^D_0_ → ^7^F_*J*_)) and total intensity involving all the ^5^D_0_ → ^7^F_*J*_ transitions (∑ ^5^D_0_ → ^7^F_*J*_), as shown in eq 1.

βR=I(5D0→7FJ)/∑D50→7FJ 2

The asymmetry of the local coordination environment around the Eu^3+^ centers can be inferred from R, which is the ratio between the integral intensities of ^5^D_0_ → ^7^F_2_ and ^5^D_0_ → ^7^F_1_ — (I^5^D_0_ → ^7^F_2_/I^5^D_0_ → ^7^F_1_)—transitions. The rationale is that the magnetic dipole ^5^D_0_ → ^7^F_1_ transition is only marginally affected by the environment and therefore its intensity is used as a reference. On the other hand, the electric-dipole-induced ^5^D_0_ → ^7^F_2_ transition is sensitive to changes in the local coordination environment. By calculating R using the intensities of the transitions, the magnitude of distortion around the Eu^3+^ ion in the polyanion can be deduced, with large *R* values indicating a strongly distorted coordination around the metal center. The short-range Judd-Ofelt (J-O) parameter Ω_2_ reflects on the local coordination environment around Eu^3+^ and the degree of covalency in the metal–ligand interaction. On the other hand, the long-range J-O parameters Ω_4_ and Ω_6_ are a manifestation of the long-range polarizability of the crystalline environment. Using the spectroscopic properties calculation module in the LUMPAC software,^15^ we have determined the values of β_*R*_, *R*, Ω_2_ and Ω_4_ from the emission spectra collected at 2.3, 77, and 300 K.

The calculation of the oscillator strengths (*f*) shed light on the relative intensities of each ^5^D_0_ → ^7^F_*J*_ transition. The values of *f* associated with the ^5^D_0_ → ^7^F_*J*_ transitions of **NaK-EuPW**_**17**_ are obtained using eq 2 and the values are collected in (Table S1).^16^

3 $$f=\frac{\varepsilon mc^{3}\xi }{2\pi n^{2}e^{2}\nu^{2}\tau }$$

where, ε is vacuum permittivity, m is mass of electron, c is the speed of light, ξ is branching ratio, *n* is refractive index, e is electronic charge, ν is frequency, and τ is lifetime. For calculating, we used *n* = 1.5, lifetime estimated for the ^5^D_0_ → ^7^F_2_ transition, and branching ratios collected in Table 4. As collected in (Table 4), the ^5^D_0_ → ^7^F_4_ transition is the most intense transition at all the temperatures. The second and third most intense ones are the ^5^D_0_ → ^7^F_4_ and ^5^D_0_ → ^7^F_1_ transitions, respectively. The small magnitude of the asymmetric ratio (Table 5), weak and strong intensities of the ^5^D_0_ → ^7^F_0_ and ^5^D_0_ → ^7^F_4_ transitions, respectively, indicate a relatively symmetric coordination environment around the Eu^3+^ center in the polyanion. The Ω_4_ > Ω_2_ indicate the operation of strong long-range effects in the crystal lattice of the compound.

**Table 4:** Temperature-dependent Branching Ratios (β_*R*_) and Oscillator Strengths (*f*) Obtained for **NaK-EuPW**_**17**_

	2.3 K		77 K		300 K	
transition	β_*R*_	*f*	β_*R*_	*f*	β_*R*_	*f*
^5^D_0_ → ^7^F_0_	0.66%	6.87 × 10^–9^	0.65%	6.87 × 10^–9^	0.59%	7.22 × 10^–9^
^5^D_0_ → ^7^F_1_	20.90%	2.27 × 10^–7^	20.43%	2.25 × 10^–7^	17.14%	2.19 × 10^–7^
^5^D_0_ → ^7^F_2_	30.56%	3.58 × 10^–7^	30.83%	3.68 × 10^–7^	36.83%	5.08 × 10^–7^
^5^D_0_ → ^7^F_3_	6.95%	9.1 × 10^–8^	7.09%	9.26 × 10^–8^	6.913%	1.06 × 10^–7^
^5^D_0_ → ^7^F_4_	40.93%	6.16 × 10^–7^	40.99%	6.27 × 10^–7^	38.52%	6.87 × 10^–7^

**Table 5:** Temperature-dependent Parameters Obtained for **NaK-EuPW**_**17**_

	temperature		
parameter	2.3 K	77 K	300 K
*R*	1.46	1.51	2.15
Ω_2_ (10^–20^ cm^2^)	2.56	2.65	3.77
Ω_4_ (10^–20^ cm^2^)	7.87	8.06	9.05
τ_rad_ (10^–3^ s)	4.23	4.13	3.47
τ_obs_ (10^–3^ s)	2.17	2.13	1.84
φ_Eu_ (%)	51.33	51.64	53.18
*k*_r_ (s^–1^)	236.56	241.97	288.42
*k*_*n*r_ (s^–1^)	224.27	226.63	253.88

The presence of one ^5^D_0_ → ^7^F_0_ transition and monoexponential lifetime decays indicate one emissive Eu^3+^ center in the polyanion. The long lifetimes in the range of 1.84 to 2.17 ms elucidate the well-shielded nature of the Eu^3+^ centers from the deactivation mechanisms such as O–H and N–H vibrations in the first coordination sphere.

To quantify the magnitude of radiative (*k*_*r*_) and nonradiative (*k*_*nr*_) decay rates involved in the emission process of **NaK-EuPW**_**17**_, we have calculated the radiative lifetime (τ_rad_) of the ^5^D_0_ state using eq 2.

4 $$\tau_{rad}={\left[A_{MD}\times {\left(n\right)}^{3}\times I_{tot}/I_{MD}\right]}^{-1}$$

where, *A*_MD_ is the spontaneous emission probability of the ^5^D_0_ → ^7^F_1_ transition in vacuum (14.65 s^–1^) and *n* is the refractive index of the polyanion (approximated as 1.45); *I*_tot_ and *I*_MD_ are the total integrated emission intensity and intensity of the magnetic-dipole-induced ^5^D_0_ → ^7^F_1_ transition, respectively. By using the calculated τ_rad_ and experimentally determined τ_obs_ (Table 5), we have estimated the rates of radiative (*k*_r_) and nonradiative (*k*_nr_) relaxation processes employing eqs 3 and 4, respectively.

5 $$k_{r}=1/\tau_{rad}$$

6 $$k_{nr}=\left[\left(1/\tau_{obs}\right)-\left(k_{r}\right)\right]$$

As collected in (Table 5), the magnitude of *k*_r_ is slightly larger than the *k*_nr_ at all the temperatures. This observation indicates that there exist quenching mechanisms causing the loss of ^5^D_0_ energy in the form of nonradiative decay. The intrinsic quantum yields (φ_Eu_) calculated employing eq 5, in the range of 51% to 53% also reflects on the nominal radiative emission efficiency of the ^5^D_0_ state.

7 $$\varphi_{Eu}=\tau_{obs}/\tau_{rad}$$

The salt **NaK-EuPW**_**17**_ comprises cocrystallized water molecules providing O–H oscillators that could cause the nonradiative deactivation of the ^5^D_0_ state. Such a possibility has been reported for Eu^3+^ complexes therefore, and hence we attribute the O–H oscillators as the reason behind the nominal intrinsic quantum yield associated with the compound. Overall, we have observed the characteristic ^5^D_0_ → ^7^F_*J*_ (*J* = 0–4) transitions upon direct excitation of the Eu^3+^ based ^7^F_0_ → ^5^L_6_ transition at 395 nm, (see Figure 13), for a summary of the emission process in the compound.

**Figure 13:**
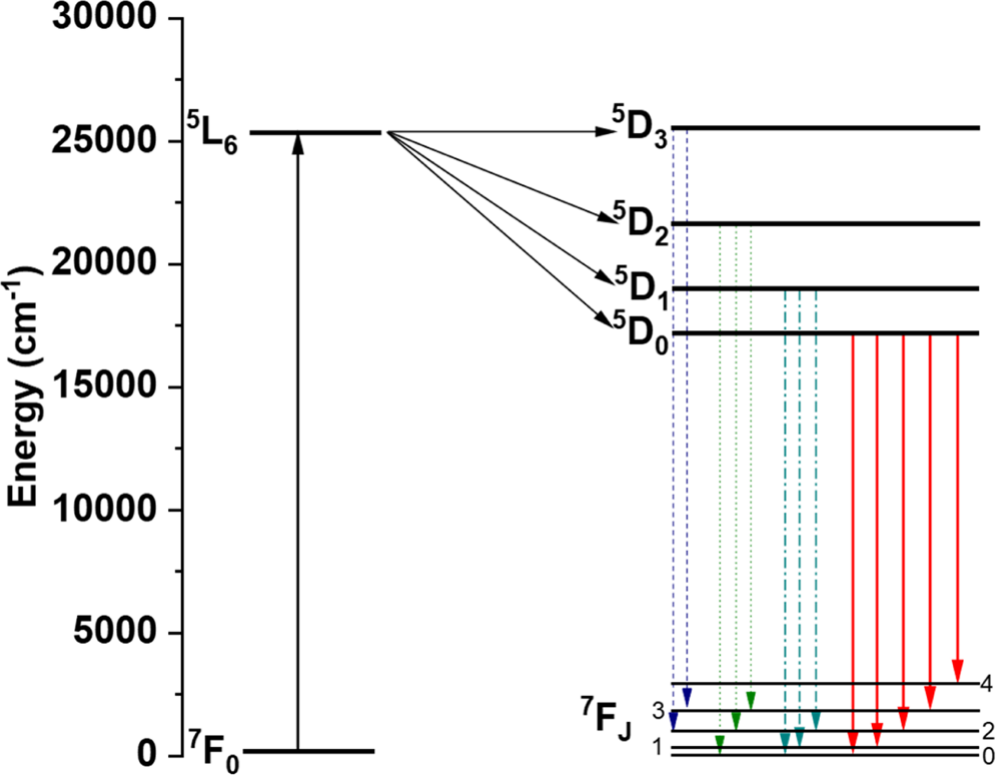
Jablonski–Perrin diagram showing the emission process in **NaK-EuPW**_**17**_. The excitation of the ^7^F_0_ → ^5^L_6_ transition at 395 nm resulted in the observation of ^5^D_0_ → ^7^F_*J*_ transition (red arrows). We have also observed ^5^D_3_ → ^7^F_*J*_ (blue dashed lines), ^5^D_2_ → ^7^F_*J*_ (green dotted lines), and ^5^D_1_ → ^7^F_*J*_ (cyan dashed-dotted lines) transitions involving the excited ^5^D_*J*_ manifolds.

The strong intensity of the ^5^D_0_ → ^7^F_4_ transition relative to the hypersensitive ^5^D_0_ → ^7^F_2_ transition indicates a symmetric coordination environment around the Eu^3+^ center. This observation is in line with the square-antiprismatic coordination geometry obtained from the SC-XRD studies discussed above. The presence of a weak yet observable ^5^D_0_ → ^7^F_0_ transition points at a distorted square-antiprismatic coordination geometry around Eu^3+^ toward a less symmetric one—for example C_2_—as discussed above. We have also studied the **YbPW**_**17**_ analogue; however, no observable Yb^3+^ transitions are noted even at 2.3 K.

Excitation of **NaK–CePW**_**17**_ at 370 nm revealed the presence of characteristic Ce^3+^ based ^2^D_3/2_ → ^2^F_5/2_ and ^2^D_3/2_ → ^2^F_7/2_ transitions at 417 nm (23981 cm^–1^) and 441 nm (22676 cm^–1^), respectively (Figure 14). An energy difference of 1305 cm^–1^ between the ^2^F_5/2_ and ^2^F_7/2_ levels is estimated.

**Figure 14:**
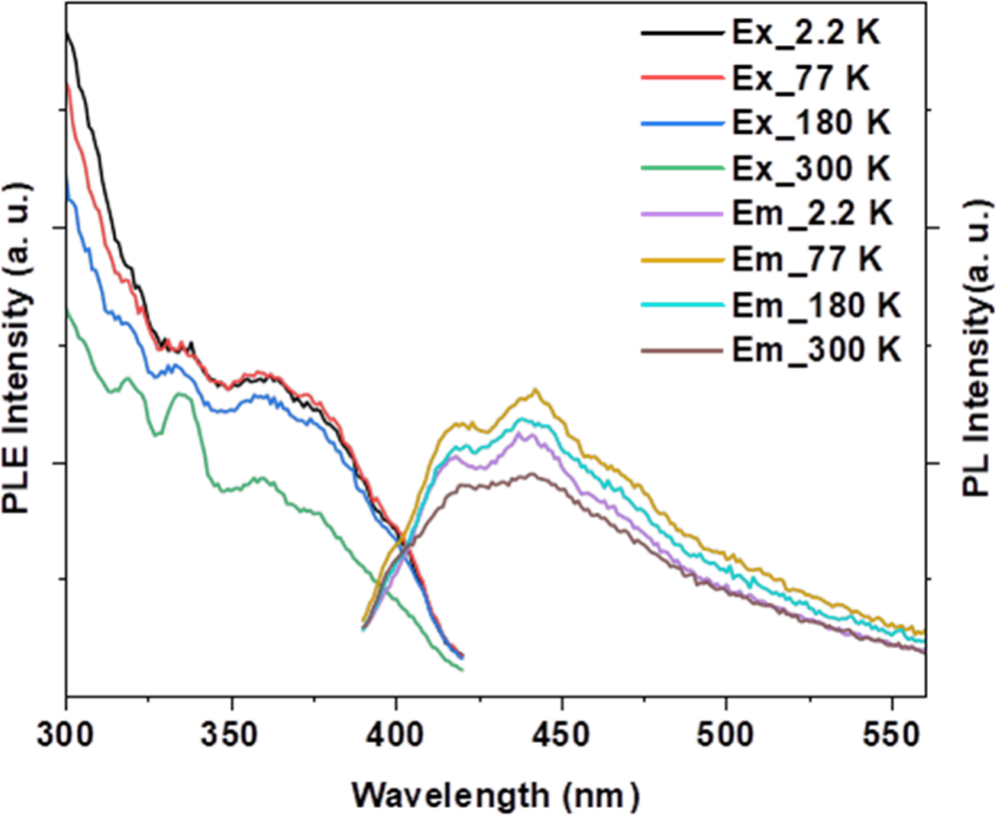
Temperature-dependent PLE and PL spectra of **NaK–CePW**_**17**_ in the solid-state. The PLE profiles were obtained by monitoring emission at 441 nm; the PL profiles were obtained by exciting the compound at 370 nm.

Remarkably, the emission intensities at 77 and 180 K are higher than the one observed at 2.2 K, the maximum intensity is observed at 77 K. Such increased intensity at intermediate temperatures, 77 and 180 K, is attributed to optimal vibronic coupling increasing the probability of emission from more vibronic levels.^17^ At 300 K, nonradiative deactivation pathways operate, hence the emission intensity decreases. The CIE coordinates (see Figure 15 and Table 6) remained almost unchanged upon temperature variation and a bluish-white emission is observed.

**Figure 15:**
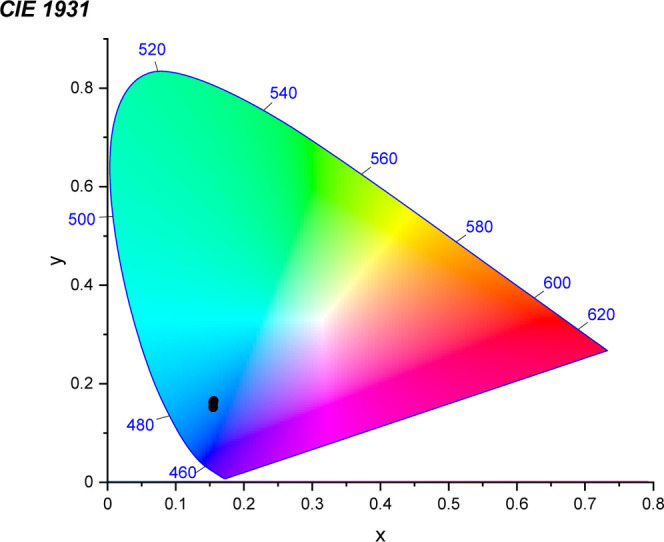
CIE 1931 diagram obtained for **NaK–CePW**_**17**_.^14^ The black dots indicate the CIE coordinates. Since the variation of the coordinates is minimal with respect to temperature, see Table 6, all the four dots are overlapped onto each other.

**Table 6:** Temperature-Dependent CIE Coordinates (*x*, *y*) Obtained for **NaK–CePW**_**17**_ ^a^

	temperature			
parameter	2.3 K	77 K	180 K	300 K
*x*	0.156	0.157	0.156	0.156
*y*	0.151	0.164	0.159	0.161

a The compound was excited at 370 nm.

## Conclusions

We have synthesized and structurally characterized an isostructural series of lanthanide-containing asymmetric Wells-Dawson-type 17-tungsto-1-phosphates with the general formula [Ln­(P­(H_4_)­W_17_O_61_)_2_]^19–^ (**LnPW**_**17**_; Ln = La^3+^, Ce^3+^, Eu^3+^, Gd^3+^, Yb^3+^, Lu^3+^, Y^3+^). As compared to the classical Wells-Dawson ion, the asymmetric derivative contains only one rather than two phosphate hetero groups and the other heteroatom site is occupied by four protons. The seven polyanions **LnPW**_**17**_ comprise a lanthanide ion being coordinated by two [P­(H_4_)­W_17_O_61_]^11–^ units in a *syn*-configuration and the lanthanide ion exhibits a square-antiprismatic coordination geometry, resulting in 8-coordination. This family of polyanions remains intact in solution, as shown by ^31^P and ^183^W NMR spectroscopy. Magnetic investigations reveal paramagnetic behavior for **NaK–CePW**_**17**_, **NaK-GdPW**_**17**_, and **NaK-YbPW**_**17**_, including field-dependent slow magnetic relaxation, underscoring their potential as functional materials in molecular magnetism. Temperature-dependent PL studies of **NaK-EuPW**_**17**_ revealed the ^5^D_0_ → ^7^F_*J*_ (*J* = 0–4) transitions upon direct excitation of the Eu^3+^-based ^7^F_0_ → ^5^L_6_ transition at 395 nm. The strong intensity of the ^5^D_0_ → ^7^F_4_ transition relative to the hypersensitive ^5^D_0_ → ^7^F_2_ transition and the presence of a weak ^5^D_0_ → ^7^F_0_ transition indicate a distorted square-antiprismatic spectroscopic site symmetry around the Eu^3+^ center, corroborating well with the all oxygen containing eight-coordinate environments observed by SC-XRD. The long ^5^D_0_ lifetime in the order of several ms elucidates the absence of deactivating pathways in the first coordination sphere of the metal center. On the other hand, the calculated intrinsic quantum yields close to 53% and the comparable magnitudes of radiative and nonradiative rate constants indicate that the cocrystallized water molecules play a crucial role in the deactivation of the ^5^D_0_ excited state by providing O–H oscillators. These findings expand the structural landscape of f-block POM chemistry and establish a versatile, reproducible route to lanthanide-functionalized hybrid frameworks.

## Supplementary Material



## Notes

FT-IR spectra,, solution ^31^P NMR and ^183^W NMR spectra, thermograms (TGA), experimental χMT­(T) data, and experimental variables used in the calculation of oscillator strengths (*f*) (PDF)
