# Supplementary material for: Reactivity of the Asymmetric Wells-Dawson Ion: Lanthanide-Containing 34-Tungsto-2-Phosphates [Ln(P(H4)W17O61)2]19– (Ln = La3+, Ce3+, Eu3+, Gd3+, Yb3+, Lu3+, Y3+)
Source: Inorg Chem. 2026 Jan 2;65(2):1051–64. doi: 10.1021/acs.inorgchem.5c04807 (PMC12820969; doi:10.1021/acs.inorgchem.5c04807)
Supplement: Supplementary file 1 [file ic5c04807_si_001.pdf]

ASSOCIATED CONTENT

Supporting Information

Reactivity of the Asymmetric Wells-Dawson Ion: Lanthanide-Containing 34-Tungsto-2-Phosphates $[Ln(P(H_4)W_{17}O_{61})_2]^{19-}$ (Ln = La^{3+}, Ce^{3+}, Eu^{3+}, Gd^{3+}, Yb^{3+}, Lu^{3+}, Y^{3+})

Mahmoud Elcheikh Mahmoud,[a] Bassem S. Bassil,[a] Anupam Sarkar,[a] Ji-o Kim,[a] Senthil Kumar Kuppusamy,[b] Concepción Molina-Jirón,[b,c,d] Eufemio Moreno-Pineda,[d,e,f] Nikoleta Malinova,[g,h] Appu Sunil,[f] Wolfgang Wernsdorfer,[b,f] Mario Ruben,[b,g,i] and Ulrich Kortz[a,]*

[a]School of Science, Constructor University, Campus Ring 1, 28759 Bremen, Germany, *Email: ukortz@constructor.university

[b]Institute of Quantum Materials and Technologies (IQMT), Karlsruhe Institute of Technology (KIT), Kaiserstraße 12, D-76131 Karlsruhe, Germany

[c] Universidad de Panamá, Facultad de Ciencias Naturales, Exactas y Tecnología, Depto. de Bioquímica, 0824, Panamá

[d] Universidad de Panamá, Facultad de Ciencias Naturales, Exactas y Tecnología, Grupo de Investigación de Materiales, Panamá, 0824, Panamá

[e] Universidad de Panamá, Facultad de Ciencias Naturales, Exactas y Tecnología, Depto. de Química-Física, 0824, Panamá.

[f] Physikalisches Institut, Karlsruhe Institute of Technology (KIT), Kaiserstraße 12, D-76131 Karlsruhe, Germany

[g] Institute of Nanotechnology (INT), Karlsruhe Institute of Technology (KIT), Kaiserstraße 12, D-76131 Karlsruhe, Germany

[h] Slovak University of Technology, Faculty of Chemical and Food Technology, Department of Inorganic Chemistry, Radlinského 2101/9, 812 37 Bratislava, Slovakia

[i] Centre Européen de Sciences Quantiques (CESQ), Institut de Science et d'Ingénierie Supramoléculaires (ISIS), 8 allée Gaspard Monge, BP 70028, 67083, Strasbourg Cedex, France

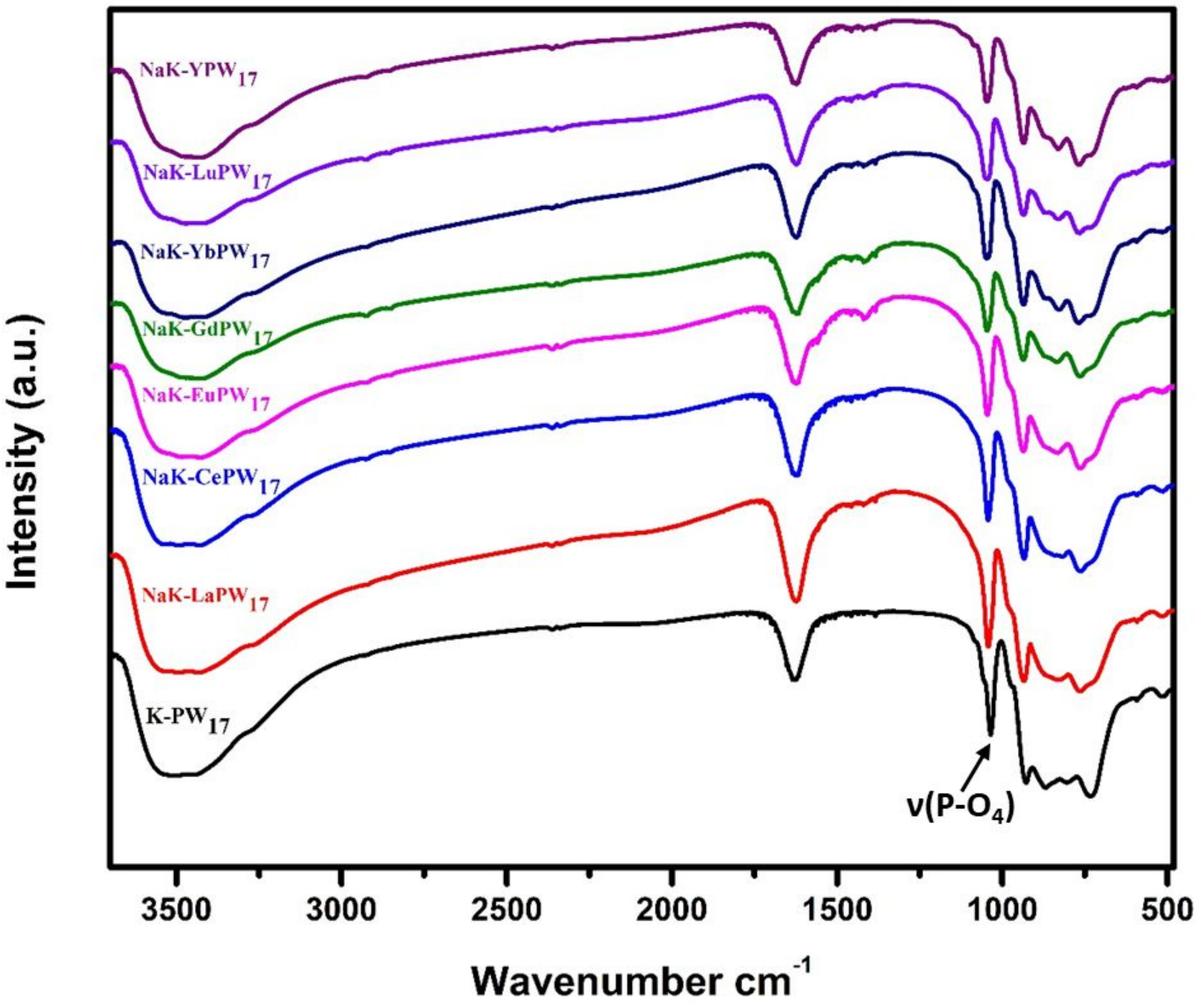

Figure S1. FT-IR spectra of **NaK-LnPW$_{17}$** (Ln = La^{3+}, Ce^{3+}, Eu^{3+}, Gd^{3+}, Yb^{3+}, Lu^{3+}, Y^{3+}) and the lacunary precursor **K-PW$_{17}$**.

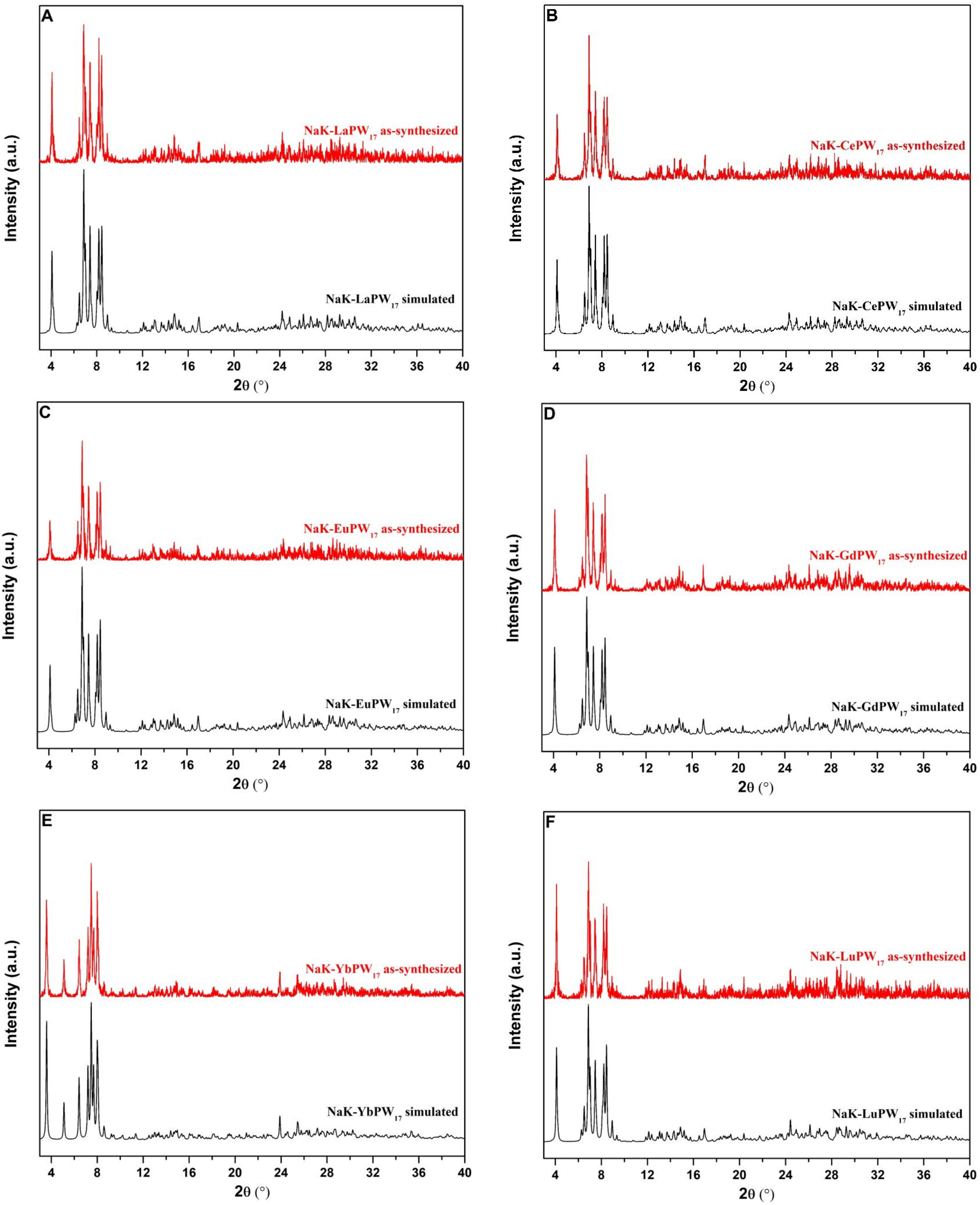
A
NaK-LaPW17 as-synthesized
NaK-LaPW17 simulated
B
NaK-CePW17 as-synthesized
NaK-CePW17 simulated
C
NaK-EuPW17 as-synthesized
NaK-EuPW17 simulated
D
NaK-GdPW17 as-synthesized
NaK-GdPW17 simulated
E
NaK-YbPW17 as-synthesized
NaK-YbPW17 simulated
F
NaK-LuPW17 as-synthesized
NaK-LuPW17 simulated
Intensity (a.u.)
2θ (°)
4
8
12
16
20
24
28
32
36
40

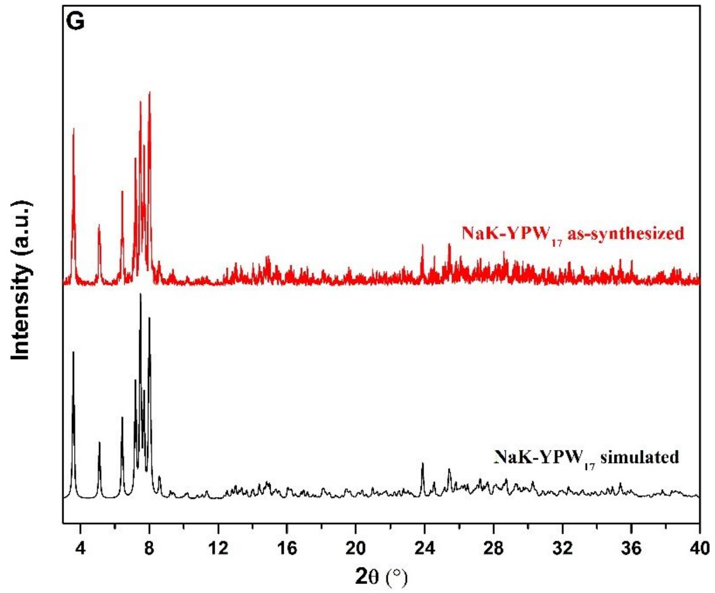

Figure S2. Powder X-ray diffraction (PXRD) patterns of the as-synthesized compounds (red) compared with the corresponding simulated patterns (black) for (A) **NaK-LaPW$_{17}$**, (B) **NaK-CePW$_{17}$**, (C) **NaK-EuPW$_{17}$**, (D) **NaK-GdPW$_{17}$**, (E) **NaK-YbPW$_{17}$**, (F) **NaK-LuPW$_{17}$**, and (G) **NaK-YPW$_{17}$**.

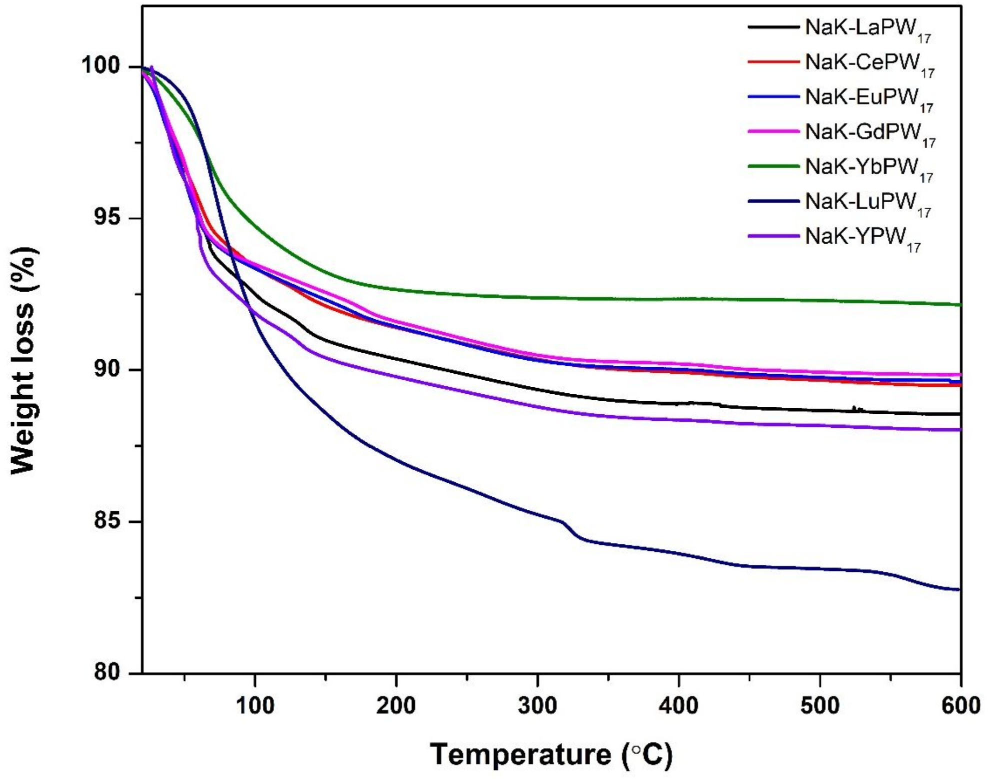

Figure S3. Thermograms of **NaK-LnPW$_{17}$** (Ln = La^{3+}, Ce^{3+}, Eu^{3+}, Gd^{3+}, Yb^{3+}, Lu^{3+}, Y^{3+}) between room temperature and 600 °C under N_2 flow.

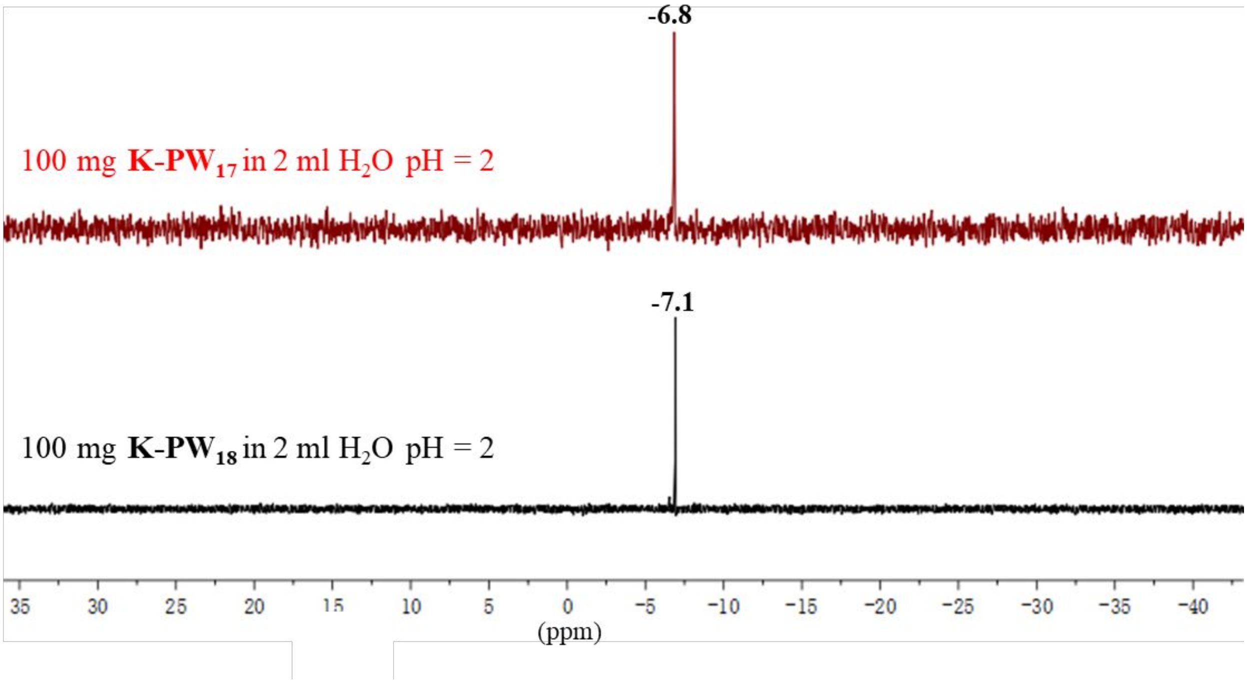

Figure S4. Room temperature ^{31}P NMR spectra for the plenary polyanion $\mathbf{PW_{18}}$ (lower) and the monolacunary derivative $\mathbf{PW_{17}}$ (upper) in water at pH 2.

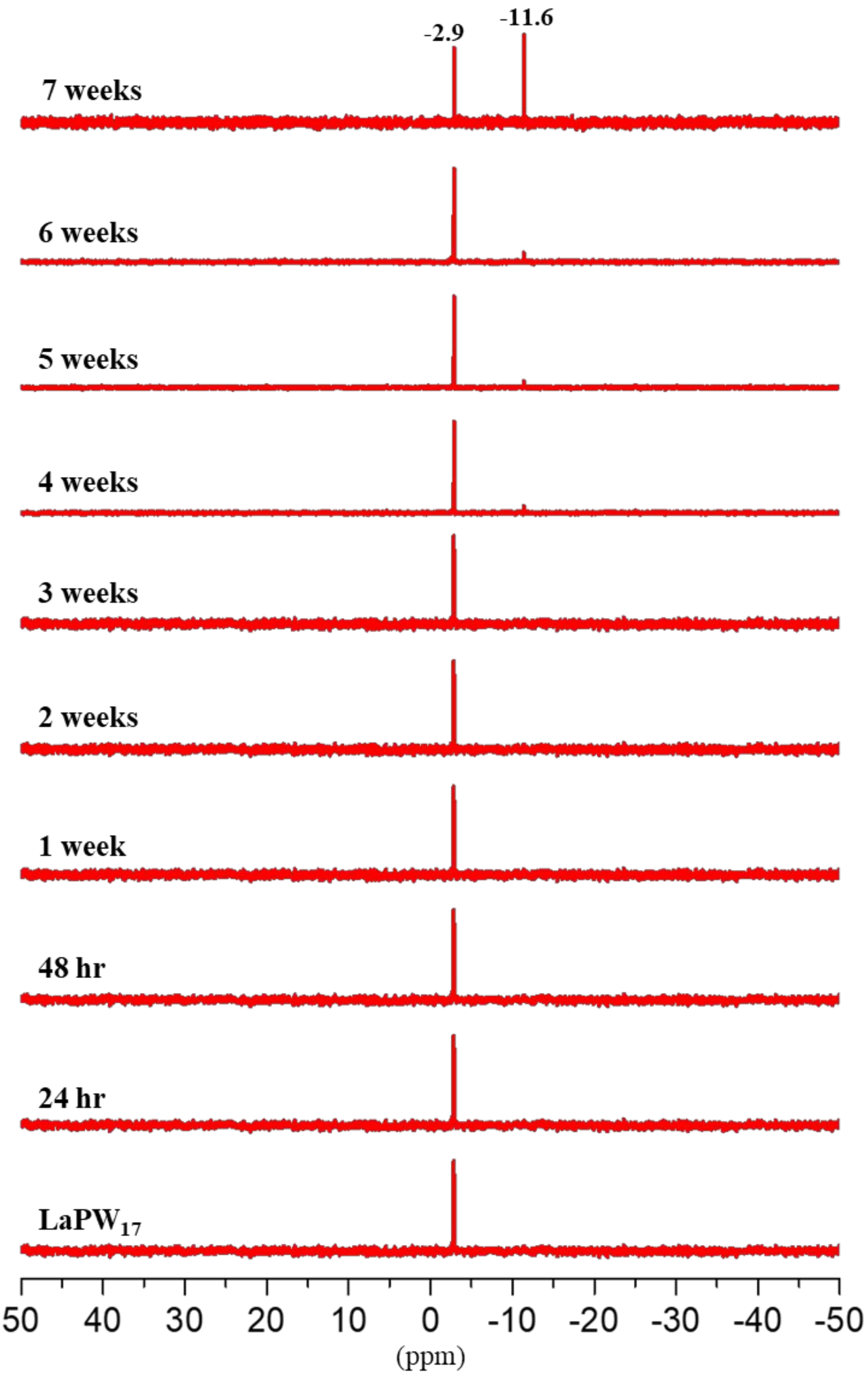

Figure S5. Time-dependent ^{31}P NMR spectra at room temperature for $\mathbf{LaPW_{17}}$ in 0.5 M CH_3COONa at pH 6.0.

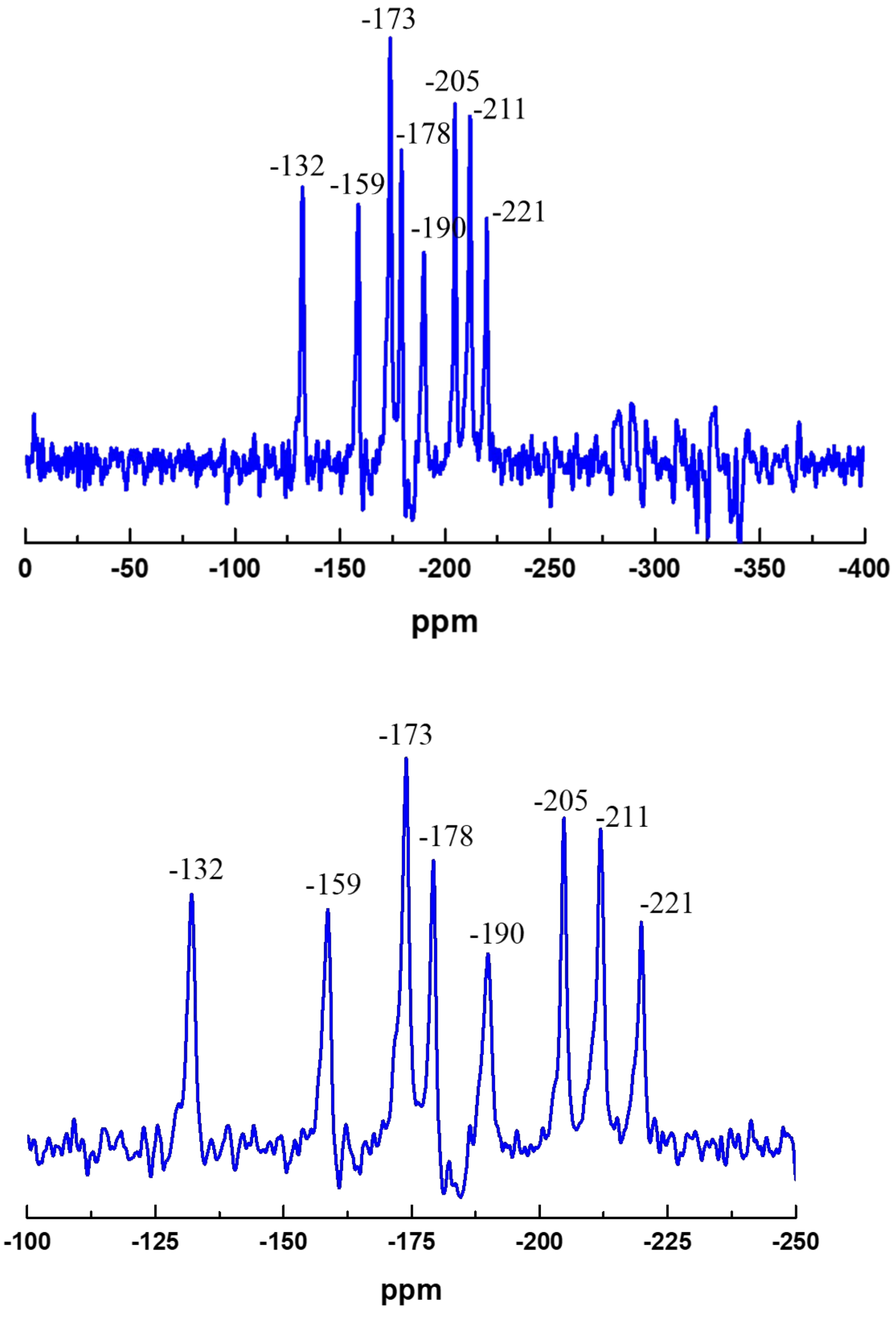

Figure S6. Room temperature ^{183}W NMR spectra of **$LaPW_{17}$** in 0.5 M CH_3COOLi at pH 6.0 from 0 to -400 ppm (upper) and -100 to -250 ppm (lower).

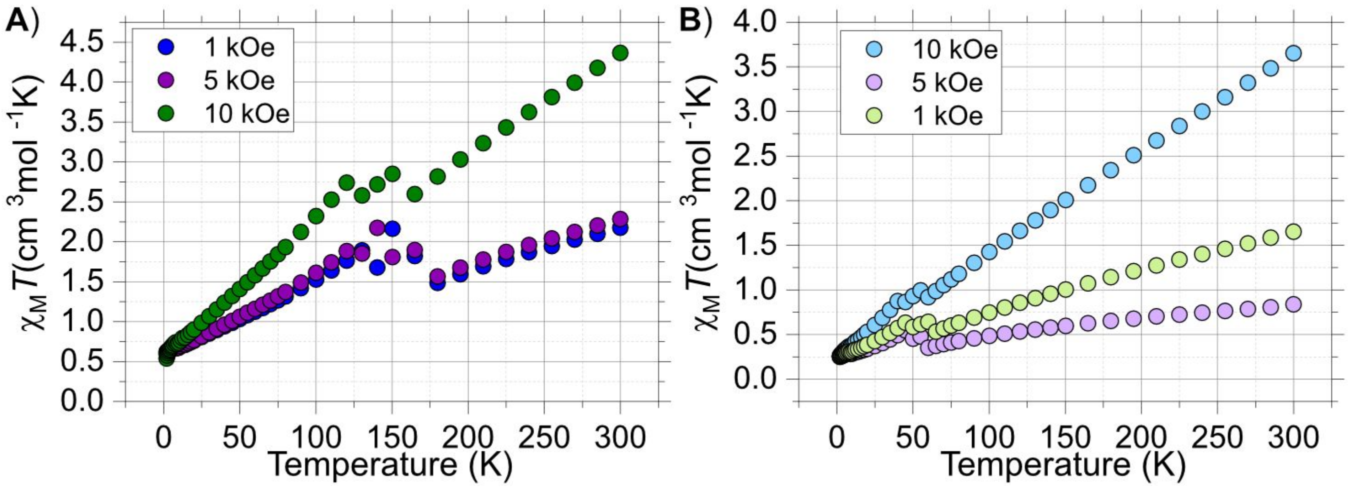

Figure S7. Experimental $\chi_M T(T)$ for (A) **NaK-CePW$_{17}$** and (B) **NaK-YbPW$_{17}$** collected at three different applied fields.

Table S1. Experimental variables used in the calculation of oscillator strengths (□) for **NaK-EuPW$_{17}$**.

Transition	2.3 K			77 K			300 K		
	ξ	τ (s)	v (10^{14} Hz)	ξ	τ (s)	v (10^{14} Hz)	ξ	τ (s)	v (10^{14} Hz)
$^5D_0 \rightarrow ^7F_0$	0.0066	0.00217	5.15	0.0065	0.00213	5.15	0.0059	0.00184	5.15
$^5D_0 \rightarrow ^7F_1$	0.2090	0.00217	5.04	0.2043	0.00213	5.04	0.1714	0.00184	5.04
$^5D_0 \rightarrow ^7F_2$	0.3056	0.00217	4.85	0.3083	0.00213	4.85	0.3683	0.00184	4.85
$^5D_0 \rightarrow ^7F_3$	0.0695	0.00217	4.59	0.0709	0.00213	4.59	0.0691	0.00184	4.59
$^5D_0 \rightarrow ^7F_4$	0.4093	0.00217	4.28	0.4099	0.00213	4.28	0.3852	0.00184	4.28
